# Evaluation of glycoprotein E subunit and live attenuated varicella‐zoster virus vaccines formulated with a single‐strand RNA‐based adjuvant

**DOI:** 10.1002/iid3.297

**Published:** 2020-03-13

**Authors:** Su Jeen Lee, Hyo‐Jung Park, Hae Li Ko, Jung Eun Lee, Hyun Joo Lee, Hun Kim, Jae‐Hwan Nam

**Affiliations:** ^1^ Department of Biotechnology The Catholic University of Korea Bucheon Republic of Korea; ^2^ Department of R&D SK Bioscience, Pangyoro Bundang‐gu Republic of Korea

**Keywords:** chickenpox, gE subunit vaccine, live attenuated vaccine, RNA adjuvant, shingles, varicella‐zoster virus

## Abstract

**Introduction:**

Varicella‐zoster virus (VZV), a human alphaherpesvirus 3, elicits both chickenpox and shingles and/or postherpetic neuralgia. A live attenuated vaccine (LAV) and glycoprotein E (gE) subunit vaccine were developed to prevent VZV‐induced diseases. We recently reported that single‐strand RNA (ssRNA) based on the intergenic region of the internal ribosome entry site of cricket paralysis virus (CrPV) is an effective adjuvant for protein‐based and virus‐like particle‐based vaccines. Here, Chinese hamster ovary expression system and an LAV from Oka/SK strains.

**Methods:**

We appraised the adjuvant effect of the same CrPV ssRNA encoding the gE gene formulated in the two vaccines using VZV‐primed C57BL/6 mice and guinea pigs. Humoral immunity and cell‐mediated immunity were assessed by enzyme‐linked immunosorbent assay (ELISA) and ELISPOT in gE subunit vaccine and by ELISA and fluorescent antibody to membrane antigen in LAV.

**Results:**

The gE subunit vaccine‐induced gE‐specific antibodies and CD4^+^ T‐cell responses (indicated by interferon‐γ [IFN‐γ] and interleukin‐2 secretion) in the ssRNA‐based adjuvant containing the VZV gE gene. Therefore, an ssRNA adjuvant combined with gE antigen can trigger the innate immune response and induce an adaptive immune response to ultimately activate humoral and cell‐mediated responses. VZV LAV could also induce VZV‐specific antibodies and IFN‐γ stimulated by LAV, whereas the effect of ssRNA as a vaccine adjuvant could not be confirmed. However, the ssRNA adjuvant increased VZV‐specific neutralizing antibody response.

**Conclusions:**

Taken together, these results highlight that the gE subunit vaccine and LAV developed in this study can be functional VZV vaccines, and ssRNAs appear to function better as adjuvants in a subunit vaccine than in an LAV.

## Graphical abstract

We evaluated two new varicella‐zoster virus (VSV) vaccines, a glycoprotein E (gE) subunit‐based vaccine and live attenuated vaccine, that were formulating with single‐strand RNA (ssRNA) as a potential adjuvant. The gE subunit vaccine and LAV developed in this study can be functional VZV vaccines, and ssRNAs appear to function better as adjuvants in a subunit vaccine than in an LAV.

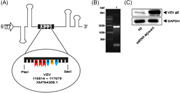

**Abbreviations**
APCs: antigen presenting cells
CHO: Chinese hamster ovary
CrPV: cricket paralysis virus
DPBS: Dulbecco's phosphate‐buffered saline
DCs: dendritic cells
ELISA: enzyme‐linked immunosorbent assay
ELISPOT: enzyme‐linked immune absorbent spot
FAMA: fluorescent antibody to membrane antigen
GAPDH: glyceraldehyde‐3‐phosphate dehydrogenase
gE: glycoprotein E
IGR: intergenic region
IFN: interferon
IL: interleukin
IRES: internal ribosome entry site
LAV: live attenuated vaccine
PBS: phosphate‐buffered saline
PHA: phytohemagglutinin
PFU: plaque forming unit
RT: room temperature
SD: standard deviations
SDS‐PAGE: sodium dodecyl sulfate polyacrylamide gel electrophoresis
ssRNA: single‐strand RNA
TLR: Toll‐like receptor
TM: transmembrane
TMB: tetramethylbenzidine
UF/DF: ultrafiltration/diafiltration
VZV: varicella‐zoster virus
WVSS: working virus seed stock

## 1 INTRODUCTION

Varicella‐zoster virus (VZV) induces chickenpox (varicella), shingles (herpes zoster), and/or postherpetic neuralgia. Varicella is the primary VZV infection and it occurs most frequently in children. Herpes zoster occurs mainly in adults or immunocompromised hosts as a consequence of latent VZV reactivation.^1^ VZV is a member of the human herpesvirus family encoding five major glycoproteins designated gpI–gpV.^2^ Glycoproteins are critical factors for VZV entry and replication. Thus, they are attractive targets for antiviral drug development.^3^ VZV gE among VZV glycoproteins is the most abundant and immunogenic. It participates in viral replication and cell‐cell transmission. Moreover, it contains B‐cell and CD4^+^ T‐cell epitopes and elicits complement‐dependent neutralizing antibodies and cell‐mediated immunity.^4^ VZV‐specific CD4^+^ T cells synthesize Th1‐like cytokines such as interleukin‐2 (IL‐2) and interferon‐γ (IFN‐γ). They induce major histocompatibility complex class II‐restricted cytotoxicity.^5^, ^6^ Therefore, CD4^+^ T cells expressing IL‐2 and IFN‐γ were selected as immune markers to evaluate cell‐mediated immune responses to VZV vaccines.^4^, ^7^, ^8^, ^9^ VZV gE is an attractive candidate for the development of VZV subunit vaccines because the VZV gE antigen, also known as CD4^+^ T‐cell antigen, is capable of inducing both humoral and cell‐mediated immune responses.^10^, ^11^, ^12^, ^13^

Vaccines currently used to prevent VZV include live attenuated vaccine (LAV) developed by Takahashi and colleagues in 1974^14^ and several other varicella vaccines licensed in several countries. The herpes zoster LAVs, Zostavax (Merck & Co., Inc., Darmstadt, Germany) and SKYZoster (SK Bioscience Co Ltd, Andong, Korea), have been licensed. LAV has preventive efficacy against varicella in the range of 70% to 96%. In contrast, its preventive efficacy against herpes zoster is only ~60%.^15^ LAV promotes relatively lower VZV‐specific cellular immune responses against herpes zoster in older patients as immunosenescence accompanies the aging process. Immunosenescence is characterized by decreased T‐cell numbers and impaired T‐cell function.^16^ A subunit vaccine comprising VZV gE and the liposome‐based adjuvant Shingrix (GlaxoSmithKline Biologicals, Rixensart, Belgium) was licensed in 2017. Its reported efficacy against herpes zoster was 97%.^15^ Therefore, the subunit vaccine may have greater efficacy against herpes zoster than LAV. A recombinant subunit vaccine is a potential alternative to live attenuated herpes zoster vaccine. However, the low immunogenicity of individual viral proteins may have to be enhanced with adjuvants.^17^

Adjuvants are immunomodulating substances that may be combined in formulations to increase their immunostimulatory efficacy.^18^ Certain adjuvants have been approved for clinical use. All new adjuvants should be compared with the gold‐standard aluminum‐based adjuvants (alum). Alum has been used to increase vaccine formulation efficacy for >90 years.^19^ However, there are still limitations of alum that require supplementation to boost vaccine efficacy. For example, alum typically induces a Th2 response, which mediates the differentiation of B cells that secrete Th2‐cell‐associated antibody isotypes, as opposed to inducing a very low Th1 response that is required to activate innate immune response,^20^, ^21^ as a prerequisite to guarantee a more effective and/or long‐term immune response.^22^ The ideal adjuvant induces innate immune responses, thereby activating adaptive immune responses.^23^, ^24^ Toll‐like receptor (TLR) agonists and oil‐in‐water emulsions have been developed to complement alum adjuvants.^25^, ^26^ Our new candidate adjuvant is a single‐strand RNA (ssRNA) derived from the cricket paralysis virus (CrPV) intergenic region (IGR) of the internal ribosome entry site (IRES). It induces balanced Th1/Th2 responses, enhances innate immune response, and increases vaccine efficacy.^27^

Here, we modified this novel ssRNA adjuvant to encode the VZV gE gene. The latter was then tested in a gE subunit vaccine expressed in a Chinese hamster ovary (CHO) cell culture platform and LAV bearing the Oka/SK strain. In VZV‐primed mice, we assessed whether this ssRNA adjuvant induces humoral‐ and cell‐mediated immunity in the VZV gE subunit vaccine. Adjuvants are not usually included in LAV formulations. However, we used guinea pigs to evaluate the ability of the ssRNA adjuvant to enhance neutralizing antibody production and cell‐mediated immune response in VZV LAV. Based on all results, we showed the potential of ssRNA adjuvants to compensate for the limitations of protein‐based vaccines with respect to low T‐cell activity and short‐term responses, as well as to increase the neutralizing antibody in LAV for preventing VZV‐induced disease.

## 2 MATERIALS AND METHODS

### 2.1 VZV gE antigen

A truncated form of the VZV gE antigen with a deleted transmembrane (TM) domain was used in this study. Gene cloning for the gE antigen was performed with a primer and a vector (pBudCE4.1) (Table 1) after isolating the DNA from Oka/SK WVSS (working virus seed stock). The VZV gE antigen was expressed in a CHO‐K1 (ATCC CCL‐61) cell line via a CHO transfection kit including Lipofectamine LTX‐Plus (No. 15338‐100; Invitrogen, Carlsbad, CA). Single‐cell colonies were obtained according to gE expression in isolated single cells. An enzyme‐linked immunosorbent assay (ELISA) identified four stable cell clone candidates with high antigen expression that were adapted in serum‐free media. After 50 serial passages, only one clone^15^ was selected. Cells with confirmed gE antigen expression were cultured to 8 L. The antigen was purified via serial ultrafiltration/diafiltration (UF/DF) steps and anion exchange chromatography and concentrated to 30 kDa for immunization. UF/DF was performed on a 10‐kDa Pellicon membrane (No. P2B010A01; Pellicon 2 Mini ultrafiltration module Biomax; EMD Millipore, Billerica, MA). Samples were loaded onto a TMAE(M) column (MiniChrom Column Fractogel® TMAE (M); Merck, Darmstadt, Germany) and eluted with 250 mM NaCl in 20 mM piperazine buffer. The eluted samples were concentrated in a 30‐kDa Centricon® Plus‐70 centrifugal filter (EMD Millipore). The final mass of the total antigen after concentration and purification was 3 mg.

**Table 1:** Primer sequences for expression vector cloning

Vector	Primers	Sequence (5→3)	Enzyme
pBudCE4.1	PB‐gE_F	TCAGGTACCCGGACCATGGGGACAGTTAAT	*Kpn*I
	PC‐gE_R	ACCGGAGGGCTTGCATGAAATAAACTCGAG	*Xho*I

### 2.2 Preparation of the RNA adjuvant

#### 2.2.1 DNA template

The RNA platform was designed with CrPV IGR IRES and SV40 late‐polyadenylation signal sequences.^28^ The RNA platform consisted of four restriction enzyme sequences and a multicloning site between the untranslated regions to permit the insertion of the VZV ORF68 (gE) gene.

#### 2.2.2 In vitro transcription and RNA purification

The DNA platform was designed with CrPV IGR IRES and SV40 late‐polyadenylation signal sequences.^28^ DNA templates were linearized with *Not*I. In vitro transcription was performed with an EZ T7 high‐yield in vitro transcription kit (Enzynomics, Seoul, Korea). Three micrograms of linearized DNA template were incubated with T7 transcription buffer, MgCl_2_, 10 mM dithiothreitol, enhancer solution, 5 mM rNTP, nuclease‐free water, and 200 units T7 enzyme mix for 1 hour at 37°C. The transcripts were incubated with RNase‐free DNase I (Promega, Madison, WI) for 15 minutes at 37°C. The reaction was terminated by incubation at 65°C for 10 minutes. RNA was purified by the LiCl method. RNA purity and concentration were evaluated with a NanoDrop 2000 spectrophotometer (Thermo Fisher Scientific, Waltham, MA). The RNA integrity was evaluated by denaturing gel electrophoresis.

#### 2.2.3 Immunoblot analysis

A549 cells were cultured in a six‐well plate for 12 hours at a density of 5 × 10^5^ per well in a medium free of 10% (v/v) fetal bovine serum. The cells were then stimulated with 2 μg ssRNA adjuvant for 24 hours, lysed by vortexing at 10‐min intervals for 1 hour in radioimmunoprecipitation assay buffer containing halt protease and phosphatase inhibitor cocktail, and centrifuged at 15 000 *g* for 30 minutes. The resulting supernatant was used as a whole‐cell lysate. Fifty‐microgram protein was loaded onto sodium dodecyl sulfate polyacrylamide gel electrophoresis gel and electrophoretically transferred to a polyvinylidene fluoride membrane. The membrane was incubated for 12 hours with the indicated VZV‐antibody (CHA Biotech, Seoul, Korea) and then incubated for 2 hours with horseradish peroxidase‐conjugated goat anti‐mouse antibody. The protein band of interest was visualized with a ChemiDoc imaging system (Bio‐Rad Laboratories, Hercules, CA). Equal protein loading was verified by glyceraldehyde‐3‐phosphate dehydrogenase immunoblotting.

### 2.3 Immunization

Six‐week‐old C57BL/6 mice were primed with VZV bulk (Oka/SK; SK Bioscience Co Ltd) at a dose of ~2000 PFU mouse^−1^. Thirty‐five days after priming, VZV gE protein (10 μg VZV antigen mouse^−1^) formulated with 20 μg ssRNA adjuvant was injected twice into the upper thigh muscles at 4‐wk intervals between inoculations. The mice were immunized in the same way with AddaVax (Cat. no. vac‐adx‐10; 10 μg; InvivoGen, San Diego, CA) as a reference control. Five groups were designated as follows: negative control (G1); LAV priming (G2); gE antigen (G3); AddaVax (G4); and ssRNA adjuvant (G5).

Six‐week‐old Dunkin‐Hartley guinea pigs were primed with VZV bulk (Oka/SK; SK Bioscience Co Ltd) at a dose of ~5000 PFU guinea pig^−1^. Thirty‐five days after priming, the guinea pigs were subcutaneously injected twice with a human dose (0.5 mL) of live attenuated herpes zoster vaccine (SKYZoster) with or without ssRNA adjuvant (50 μg) at 2‐week intervals between inoculations. Three groups were designated as follows: negative control (G1); LAV (G2); and ssRNA adjuvant (G3).

### 2.4 Immunoglobulin ELISA

VZV‐specific total immunoglobulin G (IgG), IgG1, and IgG2a in mouse serum and total IgG, IgG1, and IgG2 in guinea pig serum were measured by eELISA. The 96‐well plates (Nunc Maxisorp^TM^; Thermo Fisher Scientific) were coated with 50 ng well^−1^ VZV gE for mice and 1000 PFU well^−1^ VZV for guinea pigs and incubated overnight at 4°C. The wells were then blocked with 200 μL of 5% (v/v) skim milk for 1 hour at room temperature (RT). Diluted serum samples and VZV gE Ab (No. 127‐10031; RayBiotech, Inc, Peachtree Corners, GA) were added to the plates and incubated for 2 hours at RT. The wells were then washed three times with 200 μL phosphate‐buffered saline (PBS) mixed with 0.05% (v/v) Tween 20 (PBST). The following antibodies were then added: anti‐mouse IgG (ab97265; Abcam, Cambridge, UK), IgG1 (ab97240; Abcam), and IgG2a Ab (ab97245; Abcam) or anti‐guinea pig IgG (ab9608; Abcam), IgG1 (ABIN457757; Antibodies, Cambridge, UK), and IgG2 Ab (GAGp/IgG2/PO; Nordic MUbio, Susteren, The Netherlands). The mixtures were then incubated for 1 hour at 37°C. After washing, 3,3′,5′5′‐tetramethylbenzidine (TMB) substrate was added to the wells and the mixtures were incubated for 15 minutes. A stop solution was then added to halt the reaction. Optical densities were measured at 450 nm in a microplate reader.

### 2.5 Enzyme‐linked immune absorbent spot assay

The spleen from a mouse immunized with VZV gE antigen was washed with RPMI media and lysed with ammonium‐chloride‐potassium lysing buffer. Complete media were then added to the sample, the suspension was centrifuged for 5 minutes at 400* g*, and the pellet was washed with PBS and recentrifuged to obtain the splenocytes. One hundred microliters of the cells was loaded in each well (10^6^ cells per well). The stimulation factor phytohemagglutinin (PHA; L89025; 5 μg mL^−1^; Sigma‐Aldrich Corp, St Louis, MO) served as a positive control. Then gE protein (5 μg mL^−1^; SK Bioscience, Co Ltd, Andong, Korea) and Pepmix VZV gE (1.25 μg mL^−1^; PM‐VZV‐gE; JPT Peptide Technologies, Berlin, Germany) were added and the suspension was incubated for 20 hours at 37°C. The cells were washed with PBST and anti‐murine IFN‐γ and IL‐2 biotin detection Ab (ImmunoSpot® murine IFN‐γ and IL‐2 single‐color enzymatic enzyme‐linked immune absorbent spot (ELISPOT) kit; Cellular Technology Ltd., Shaker Heights, OH) was added to each well. The plate was incubated for 2 hours at RT and washed three times with 200 μL PBST. STREP‐Ab solution from the aforementioned kit was diluted to 1:1000. Then 80 μL of this dilution was added to each well and the suspensions were incubated for 30 minutes at RT. Eighty microliters of diluted substrate solution was then added to each well. The wells were rinsed with distilled water to stop the reaction, the plates were allowed to air‐dry, and the spots were counted.

### 2.6 Cytokine ELISA

Cultured guinea pig splenocytes (10^6^) harvested at 18 days after the second immunization with live VZV bulk containing Oka/SK strains and ssRNA adjuvant were mixed with a live virus (1000 plaque‐forming unit, PFU) and PHA (5 μg mL^−1^). The cells were incubated for 19 hours at 37°C and plated in a 96‐well plate coated with antibodies against IFN‐γ (Guinea pig Interferon‐γ ELISA Kit; abx051108; Abbexa Ltd, Cambridge, UK) or IL‐2 (Guinea pig IL‐2 ELISA Kit; abx150425; Abbexa Ltd). The standards and samples were added to the wells and the suspensions were incubated and washed with wash buffer in the kit. One hundred microliters of biotin‐conjugated antibody against IFN‐γ was used for detection. After washing, 90 μL TMB substrate was added to the wells. The suspensions were incubated for 30 minutes at 37°C to visualize horseradish peroxidase activity. To stop the reaction, 50 μL stop solution was added to each well. Optical densities were measured in a microplate reader at 450 nm to calculate the IFN‐γ or IL‐2 concentration.

### 2.7 Fluorescent antibody to membrane antigen assay

Fluorescent antibody to membrane antigen (FAMA) was used to measure the production of neutralizing antibodies to VZV. To determine the anti‐VZV IgG level, 30 μL Dulbecco's phosphate‐buffered saline (DPBS) was added to U‐bottom 96‐well plates. Guinea pig serum was serially diluted from 1:2 to 1:1024. Cell‐associated virus (30 μL) from infected cells was added to the wells and the suspensions were incubated for 30 minutes at 37°C. After centrifugation at 2000 rpm for 5 minutes, the supernatant was removed and the cells were washed with 1% (v/v) gelatin‐DPBS (2:1) buffer. Then 30 μL of a 1:200 dilution of anti‐GP IgG‐fluorescein isothiocyanate conjugate was added to each well and the suspensions were incubated for 30 minutes at 37°C. After washing with 1% (v/v) gelatin‐DPBS buffer, 4 μL glycerol‐DPBS (2:1) was added to each well and the suspensions were visualized by fluorescence microscopy.

### 2.8 Statistical analysis

All histomorphometry data are expressed as means ± standard deviations (SD). One‐way analysis of variance was run to compare group means. A Levene test was performed to evaluate variance homogeneity. If no significant deviations were found, the data were then analyzed by the least significant difference test. If the Levene test detected significant deviations from variance homogeneity, the data were then assessed by a Bonferroni test. Statistical analyses were conducted in SPSS v. 14.0 for Windows (release 14.0 K; IBM Corp, Armonk, NY). Student *t* test was run to identify differences between groups with large errors. Differences were considered significant at *P* < .05.

## 3 RESULTS

### 3.1 VZV gE antigen preparation

**Figure 1:**
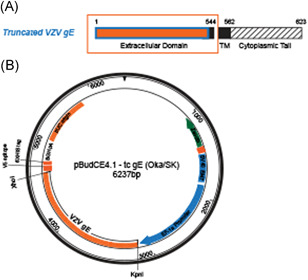
Cloning of truncated VZV gE. A, Truncated VZV gE contained 544 of the 623 amino acids present in the full‐length protein. In the former, the TM and CT regions were deleted. B, Cloning expression vector (pBudCE4.1‐tc‐gE). Truncated VZV gE (1632 bp) was inserted into the pBudCE4.1 vector with *Kpn*I*‐Xho*I. CT, cytoplasmic tail; gE, glycoprotein E; VZV, varicella‐zoster virus

**Figure 2:**
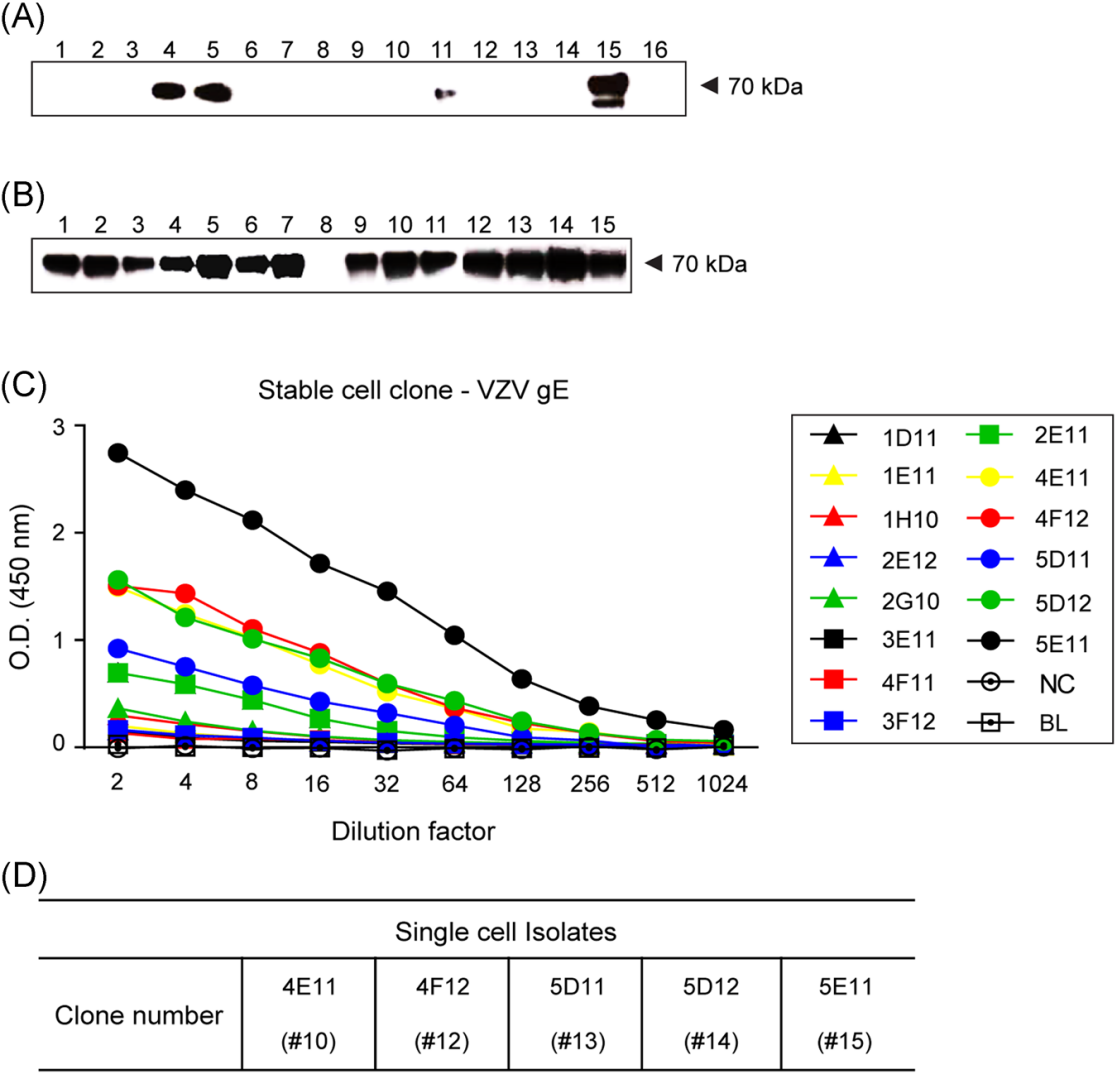
Confirmed VZV gE antigen expression (~70 kDa) by clones in CHO‐K1 cells. A, Clones 4, 5, 11, and 15 expressed gE antigen in the first single‐cell cloning. B, Clones derived from clone 5 in (A) expressing gE antigen in second single‐cell cloning. C, ELISA results of 14 clones in (B). D, Five clones showed the highest ELISA detection from (C). Lane numbers indicate each separately cultured clone. CHO, Chinese hamster ovary; CT, cytoplasmic tail; gE, glycoprotein E; TM, transmembrane; VZV, varicella‐zoster virus

To prepare the VZV gE antigen for mouse immunization, we isolated the ORF68 gene (gE + gpI) from Oka/SK WVSS (SK Bioscience Co Ltd). The full‐length gE gene was 1872 bp and it encoded 623 amino acids. However, the truncated form used for gE antigen expression here was 1632 bp and it encoded 544 amino acids. In the latter case, the transmembrane and the cytoplasmic tail (CT) of the *C*‐terminal were deleted (Figure 1A).^29^ The cloned truncated gE genes (Figures 1B and S1) were expressed in CHO‐K1 cells. A Western blot analysis of the purified VZV gE proteins confirmed the expression of clones 4, 5, 11, and 15 in the first single cells (Figures 2A and S2) and in 14 clones derived from clone 5 (Figure 2B). ELISA revealed that clones 10, 12, 13, 14, and 15 had high antigen expression (Figure 2C,D). Thus, they were selected for suspension culture adaptation in a serum‐free medium.

### 3.2 LAV preparation

The LAV used to immunize the guinea pigs was manufactured by SK Bioscience Co Ltd. It was approved in 2017 under the name SKYZoster and prescribed as a prophylactic against shingles (zoster) for adults aged ≥50 years. Its active ingredient is Oka/SK strain and the other constituents include stabilizers such as gelatin, sucrose, and urea. The master‐ and working virus seeds (Oka/SK) were established with the attenuated Oka vaccine strain. Vaccine safety and immunogenicity were validated in clinical phase II/III trials and compared against Zostavax (Merck & Co Inc, Whitehouse Station, NJ).^30^ One lot from the commercial products (≥27 400 PFU 0.5 mL^−1^) manufactured by SK Bioscience Co Ltd was used for the immunization here.

### 3.3 Humoral immune response against VZV gE antigen in mice

**Figure 3:**
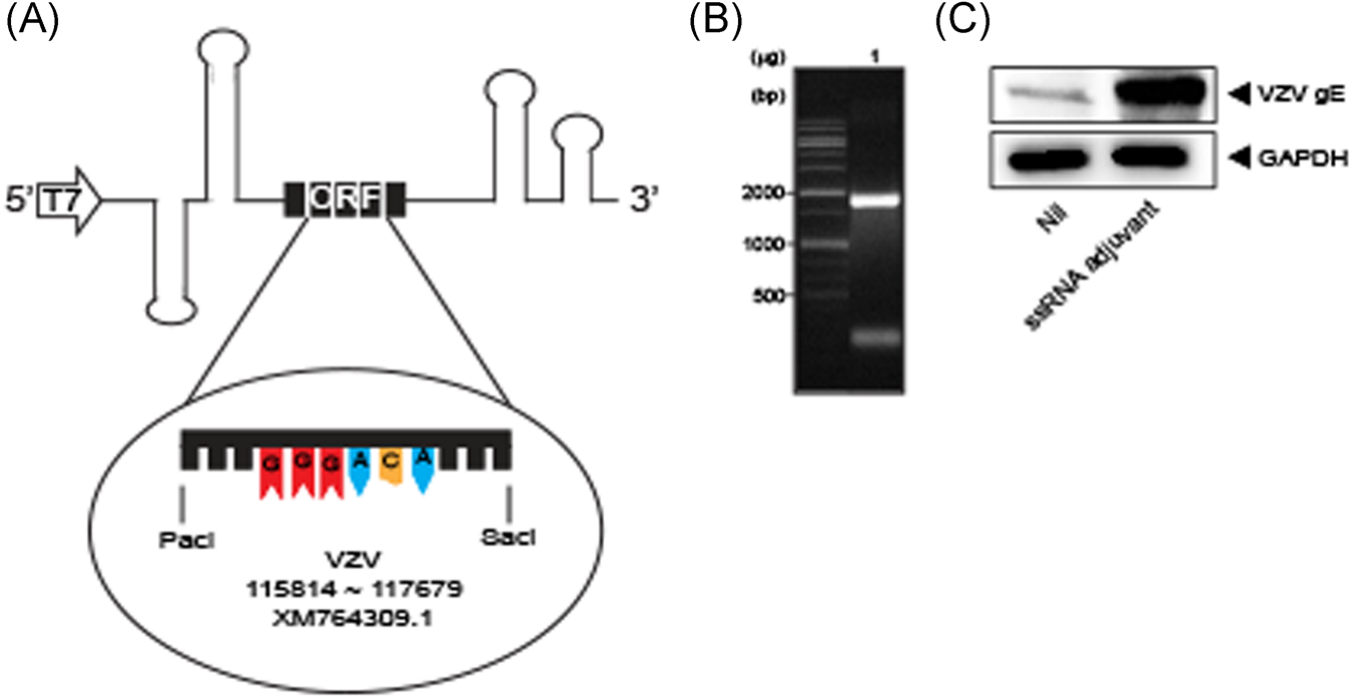
Structure of ssRNA adjuvant encoding VZV gE gene. A, Schematic of ssRNA adjuvant encoding VZV gE gene. B, Denaturing gel electrophoresis of ssRNA adjuvant. C, Immunoblot of VZV gE gene in A549 cells transfected with ssRNA adjuvant (2 μg) for 24 hours. GE, glycoprotein E; ssRNA, single‐strand RNA; VZV, varicella‐zoster virus

The ssRNA adjuvant was prepared using the RNA platform derived from CrPV IGR IRES (Figure 3A) and the VZV ORF68 gene (gE). *PacI‐SacI* was used in the gene of interest (Figure 3B). Western blot with gE antibody (CHA Biotech, Seoul, Korea) confirmed that the ssRNA adjuvant with VZV ORF68 gene expressed 68‐kDa gE protein in transfected A549 cells (Figure 3C).

**Figure 4:**
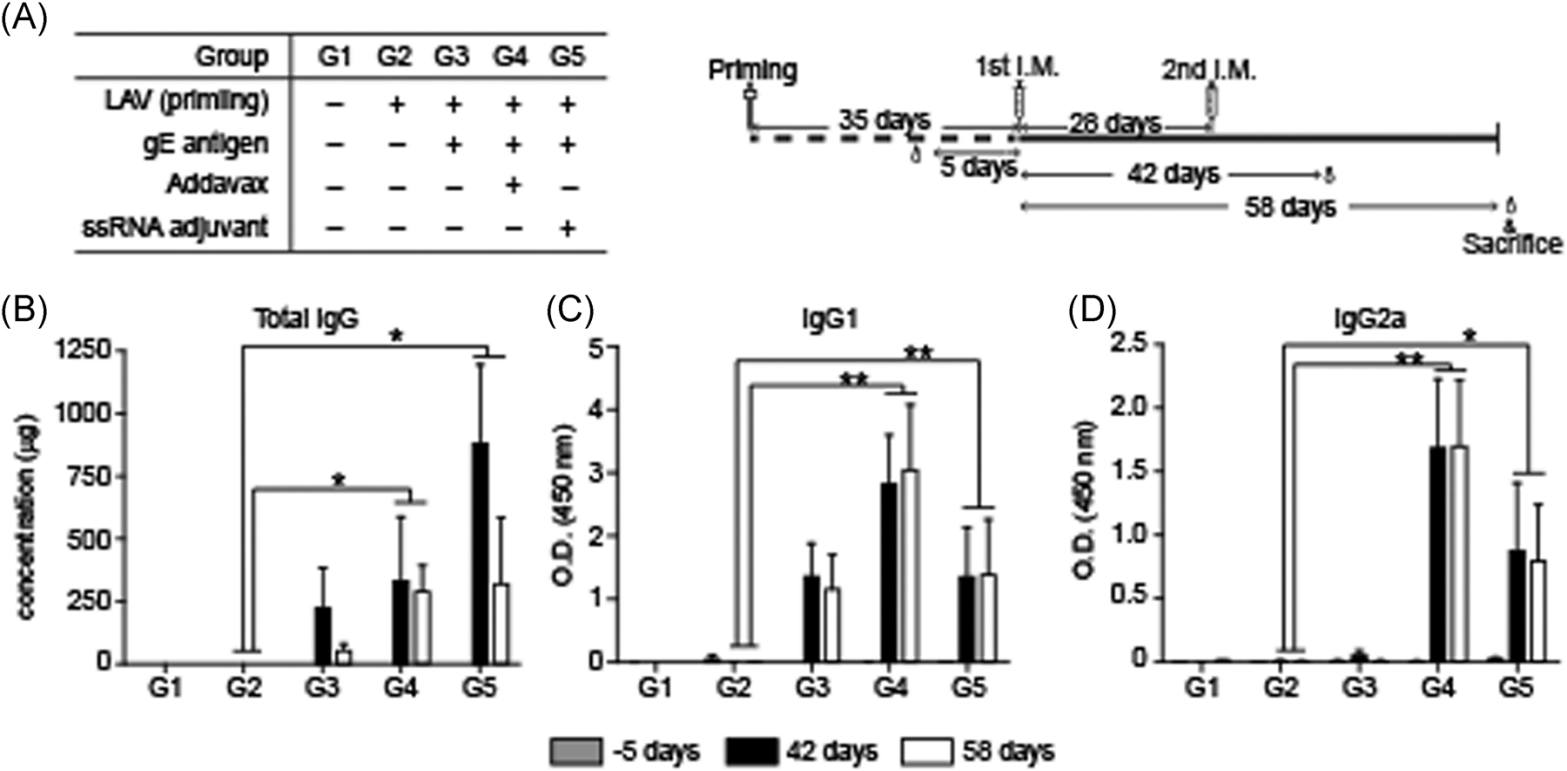
Humoral immune response of VZV‐gE induced in C57BL/6 mice. A, Study design for gE protein‐based vaccine immunization and schedules. All groups were inoculated twice at 4‐wk interval. Sera at pre‐injection (Day ‐5), 14 days (Day 42), and 30 days (Day 58) after second immunization were analyzed. Total IgG (B), IgG1 (C), and IgG2a (D) titers at –5 days, 42 days, and 58 days after first immunization. Data are means ± SD. gE, glycoprotein E; I.M., intramuscular; IgG, immunoglobulin G; LAV, live attenuated vaccine; O.D. optical density; VZV, varicella‐zoster virus. **P < .01, *P < .05 compared with LAV priming group G2

To confirm the immune responses in mice subjected to the ssRNA adjuvant with the VZV gE gene, mouse blood was sampled for IgG detection at 5 days before the first immunization and again at 42 and 58 days after the first immunization (Figure 4A). Total IgG, IgG1, and IgG2a titers were all similar at Days 42 and 58 for nearly all mouse groups. The VZV gE total IgG antibody titers were higher in G5 (with ssRNA adjuvant) than G3 (without ssRNA adjuvant) (Figure 4B). The IgG1 titers indicating a Th2 response were similar for G5 and G3 (Figure 4C) whereas the IgG2a titer indicating a Th1 response for G5 was about 10‐fold higher than that for G3 (Figure 4D). G4 (with AddaVax) presented with the highest IgG1 and IgG2a titers (Figure 4C,D).

### 3.4 CD4^+^ T‐cell‐mediated immune response against VZV gE antigen in mice

**Figure 5:**
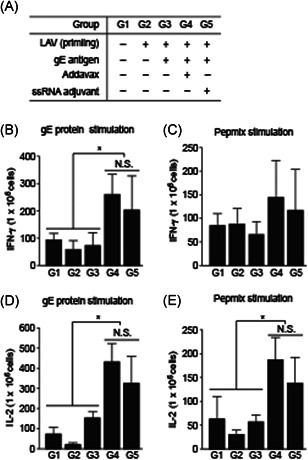
Cytokine‐secreting cell frequencies in immunized mouse splenocytes. IFN‐γ‐secreting cell frequency in immunized mouse splenocytes after stimulation with gE protein (A) and Pepmix (B). IL‐2‐secreting cell frequency in immunized mouse splenocytes after stimulation with gE protein (C) and Pepmix (D). Data are means ± SD. gE, glycoprotein E; IFN‐γ, interferon‐γ; IL‐2, interleukin‐2; N.S., no significance. *P < .05

VZV‐specific cytokines (IFN‐γ and IL‐2; also known as CD4^+^ T‐cell cytokines)^5^ were measured by ELISPOT assay to confirm enhancement of the VZV‐specific cellular immune response^31^, ^32^ with splenocytes from immunized mice at day 58 after the first immunization. The numbers of IFN‐γ‐ and IL‐2‐secreting cells in the cultured splenocytes had increased in G5 (with ssRNA adjuvant) compared with those in G3 (without ssRNA adjuvant) after stimulation with gE protein and gE‐specific peptide mixture (Pepmix) (Figure 5A‐D). Moreover, the numbers of cells secreting IFN‐γ and IL‐2 were similar in G5 (with ssRNA adjuvant) and G4 (with Addavax) used as the reference control (Figure 5A‐D). The numbers of IFN‐γ‐ and IL‐2‐secreting cells after PHA stimulation (as a positive control) were dramatically increased in all groups (Figure S3(A) and (b)).

### 3.5 Humoral and CD4^+^ T‐cell‐mediated immune responses against LAV for VZV in guinea pig

**Figure 6:**
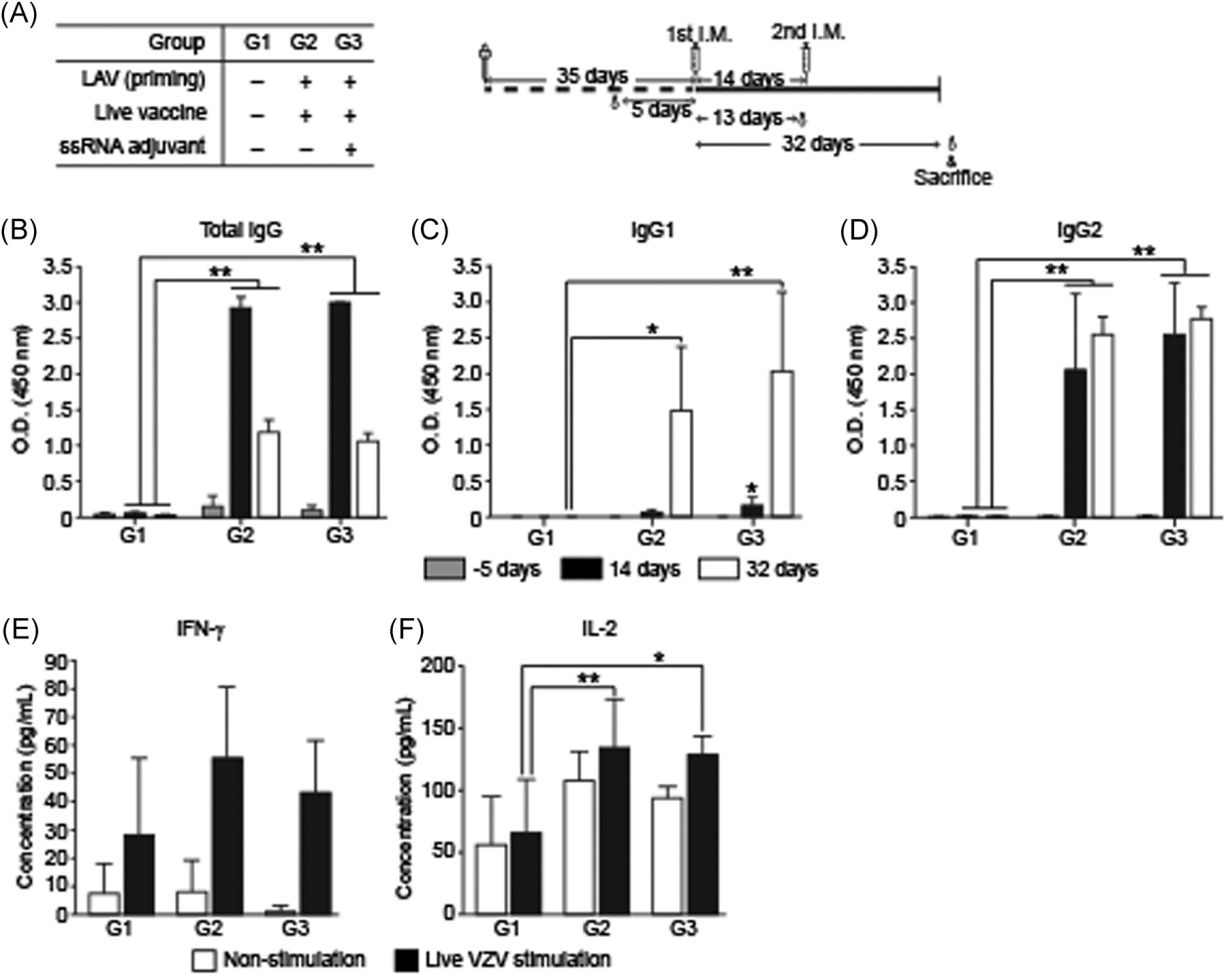
Humoral immune responses and IFN‐γ and IL‐2 levels in guinea pigs immunized with LAV. A, Study design for VZV LAV immunization and schedules. All groups were inoculated twice at 2‐week intervals. Sera at pre‐injection (Day ‐5), 14 days, and 32 days after the first immunization were analyzed for total IgG (B), IgG1 (C), and IgG2 (D). IFN‐γ (E) and IL‐2 (F) levels in supernatants of cultured splenocytes after stimulation with LAV were measured by cytokine ELISA. Data are means ± SD. ELISA, enzyme‐linked immunosorbent assay; IFN‐γ, interferon‐γ; IL‐2, interleukin‐2; IgG, immunoglobulin G; LAV, live attenuated vaccine.** *P* < .01, *P < .05 compared with PBS immunization group G1

We tested the effects of ssRNA adjuvant in LAV SKYZoster (SK Bioscience Co Ltd) which was approved for administration as a shingles vaccine. Blood samples were obtained at 5 days before the first immunization and again at 14 and 32 days after the first immunization (Figure 6A). Total IgG, IgG1, and IgG2 titers were not significantly increased in G3 (with ssRNA adjuvant) relative to those in G2 (without ssRNA adjuvant) (Figure 6B‐D). Thus, the ssRNA adjuvant had no influence on the live vaccine. IgG1 (Th2 immune response) showed a weak titer at 14 days after the first immunization but was dramatically increased after the second immunization at Day 32 in all groups (Figure 6C).

CD4^+^ T‐cell‐mediated immune response was assessed by measuring IFN‐γ and IL‐2 in cultured splenocyte supernatants after VZV stimulation. The VZV‐induced cytokines IFN‐γ and IL‐2 were slightly increased in G2 (without ssRNA adjuvant) and G3 (with ssRNA adjuvant) compared to that in G1 (negative control). However, the cytokine levels were not significantly different between G2 and G3 (Figure 6E,F). As a positive control, the numbers of IFN‐γ‐secreting cells after PHA stimulation were increased in all groups (Figure S4A). But the numbers of IL‐2‐secreting cells were not increased significantly after PHA stimulation (Figure S4B). Therefore, the ssRNA adjuvant was ineffective in LAV.

### 3.6 Neutralizing antibodies against VZV in guinea pigs

**Figure 7:**
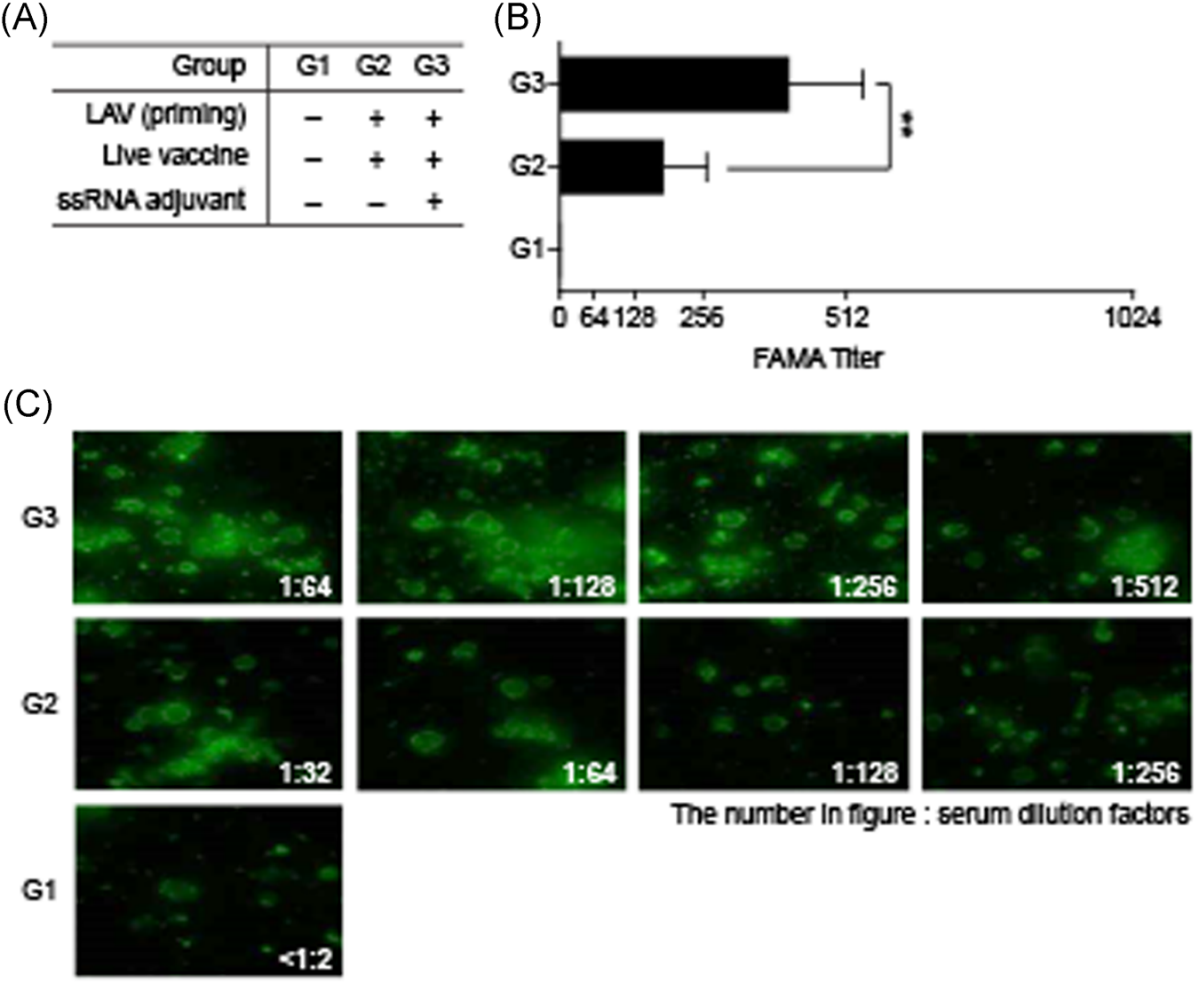
Neutralizing antibodies analyzed by FAMA to determine boosting effect of ssRNA adjuvants on VZV LAV. Study design for VZV LAV immunization is shown in Figure 6A. Guinea pig serum was serially diluted from 1:2 to 1:1024. Anti‐GP IgG‐FITC conjugate was used with 1:200. A, Average FAMA titer was 186 for G2 (LAV alone) and 410 for G3 (LAV + ssRNA). B, Cells were observed under fluorescence microscope. FAMA, fluorescent antibody to membrane antigen; FITC, fluorescein isothiocyanate; IgG, immunoglobulin G; LAV, live attenuated vaccine; ssRNA, single‐strand RNA; VZV, varicella‐zoster virus

VZV‐specific neutralizing antibodies were measured by FAMA. The titers in G3 (with ssRNA adjuvant) were about 2‐fold higher than those in G2 (without ssRNA adjuvant) (Figure 7A,B). Therefore, the ssRNA adjuvant‐induced neutralizing antibodies and, by extension, a humoral immune response in the LAV to the same level as the protein subunit vaccine even without corresponding increases in ELISA antibodies or cytokines.

## 4 DISCUSSION

ssRNA is recognized by TLR7 and/or TLR8, which are highly expressed in antigen‐presenting cells (APCs) to stimulate an adaptive immune response for antigen‐specific T cells, accompanied by high‐affinity antibody production.^25^ Despite this potential adjuvant effect, to date, RNA has not been applied in conventional vaccines. Here, we produced a VZV gE subunit vaccine and a LAV containing the Oka/SK strain and integrated our recently developed CrPV IGR IRES‐derived ssRNA adjuvant encoding the VZV gE gene into them.

Mice immunized with the subunit vaccine containing the ssRNA adjuvant responded with IgG1 (Th2 response) and IgG2a (Th1 response) titers that were higher than those for the groups without ssRNA adjuvant. The numbers of IFN‐γ‐ and IL‐2‐secreting cells were 2 to 3‐fold higher in the ssRNA adjuvant‐immunized groups than in those immunized with VZV gE antigen alone. IFN‐γ and IL‐2 activate Th1 cells that enhance pro‐inflammatory, cell‐mediated immunity.^33^ Increases of the IL‐2‐secreting immune cells indicates elevated T‐cell activation, expansion, differentiation, and maintenance. It also indicates the differentiation of CD8^+^ T cells into terminal effector‐ and memory cells.^34^, ^35^ Induction of VZV‐specific T‐cell activation with ssRNA adjuvant is essential for the development of VZV vaccines for administration to older patients because T‐cell activity declines with age.^4^ Thus, ssRNA adjuvant may play an important role in VZV‐specific T‐cell immunity and enhance cell‐mediated immune response to vaccination.

Our data demonstrated that the ssRNA adjuvant boosts gE antigen immunogenicity and augments gE‐specific antibody and CD4^+^ T‐cell responses. Therefore, it enhances cell‐mediated‐ and humoral immune responses. These findings corroborate those of a previous study in which the ssRNA adjuvant was formulated with Middle East respiratory syndrome coronavirus spike protein.^27^ Protein‐based vaccines are relatively less immunogenic because the potency of T‐cell activation is low.^36^ Thus, the ssRNA adjuvants must compensate for these deficiencies. The gE protein produced from the CHO‐K1 cell expression system‐induced VZV gE‐specific antibody. Thus, it is a good candidate for VZV protein vaccine.

The ssRNA adjuvant in LAV (SKYZoster) did not increase humoral‐ or cellular immune responses in guinea pigs. LAVs can generally initiate innate immunity in dendritic cells (DCs). DCs induce innate inflammatory responses to pathogens in a manner similar to responses to viral infections. These innate immunity responses drive subsequent adaptive responses such as memory T‐ and B‐cell activation.^37^, ^38^, ^39^ Adjuvants may function as pathogen‐associated molecular patterns that trigger innate immune responses via different mechanisms including APC activation and maturation and the initiation of downstream adaptive immune activity.^40^ In an earlier study, we showed that ssRNA adjuvant‐induced immune response‐related genes similar to those induced by LAV.^27^ Therefore, elicitation of the innate and adaptive immune responses by ssRNA adjuvant may be offset by the effects of VZV LAV.

The neutralizing antibodies measured by FAMA were about two‐fold higher in the groups receiving the ssRNA adjuvant in VZV LAV than in those that did not receive it despite the lack of difference in ELISA antibody (IgG1 and IgG2a) titers or Th1‐related cytokine induction. The ssRNA adjuvant must recruit comparatively more APCs (especially DCs) at the injection site^27^ and the resultant increase in APCs may present specific epitopes to B cells. However, further research is needed to validate this hypothesis.

AddaVax was the reference control vaccine used in this study. It resembles MF59 and is a squalene‐based oil‐in‐water nanoemulsion that enhances cellular‐ and humoral immune responses. In contrast, AlhydroGel (aluminum hydroxide gel) drives a Th2 response. AddaVax significantly increases antibody titers with more balanced Th1/Th2 responses than those obtained with alum.^41^ Here, the ssRNA adjuvant‐induced humoral and balanced Th1/Th2 immune responses even though its antibody and cytokine induction levels in the VZV gE protein subunit vaccine were lower than those for AddaVax. Nevertheless, the mode of action of AddaVax has not yet been elucidated.^42^, ^43^ A previous study established that ssRNA adjuvants are relatively safe.^44^

Taken together, these results verify that the gE protein produced in this study can be a new VZV protein vaccine candidate. Moreover, the ssRNA derived from CrPV IGR IRES encoding the gE gene, which could express gE in transfected cells, can effectively function as a vaccine adjuvant in a protein‐based subunit vaccine by activating humoral and cell‐mediated immune responses. These findings are in accordance with our previous report.^27^ In addition, we showed that the ssRNA adjuvant could boost the production of neutralizing antibodies even in an LAV. Thus, it is expected that this previously developed ssRNA adjuvant will be useful as an immune stimulator for various vaccine types, including protein‐based and even LAVs.

## CONFLICT OF INTERESTS

The authors declare that there are no conflict of interests.

## ETHICS STATEMENT

All immunization‐related animal experiments in this study were conducted in accordance with the guidelines of the Institutional Animal Care and Use Committee of the QuBEST BIO (Approval No. QBSIACUC‐A18090 for mouse/QBIACUC‐A18024 for guinea pig).

## Supporting information

Supporting information

Supporting information

Supporting information

Supporting information

Supporting information

## Data Availability

All data are shown within the manuscript and figures. The raw data and the analysis details that support the findings of this study are available from the corresponding author upon reasonable request.
