# Supplementary figures and images for: Evaluation of glycoprotein E subunit and live attenuated varicella‐zoster virus vaccines formulated with a single‐strand RNA‐based adjuvant
Source: Immun Inflamm Dis. 2020 Mar 13;8(2):216–27. doi: 10.1002/iid3.297 (PMC7212201; doi:10.1002/iid3.297)

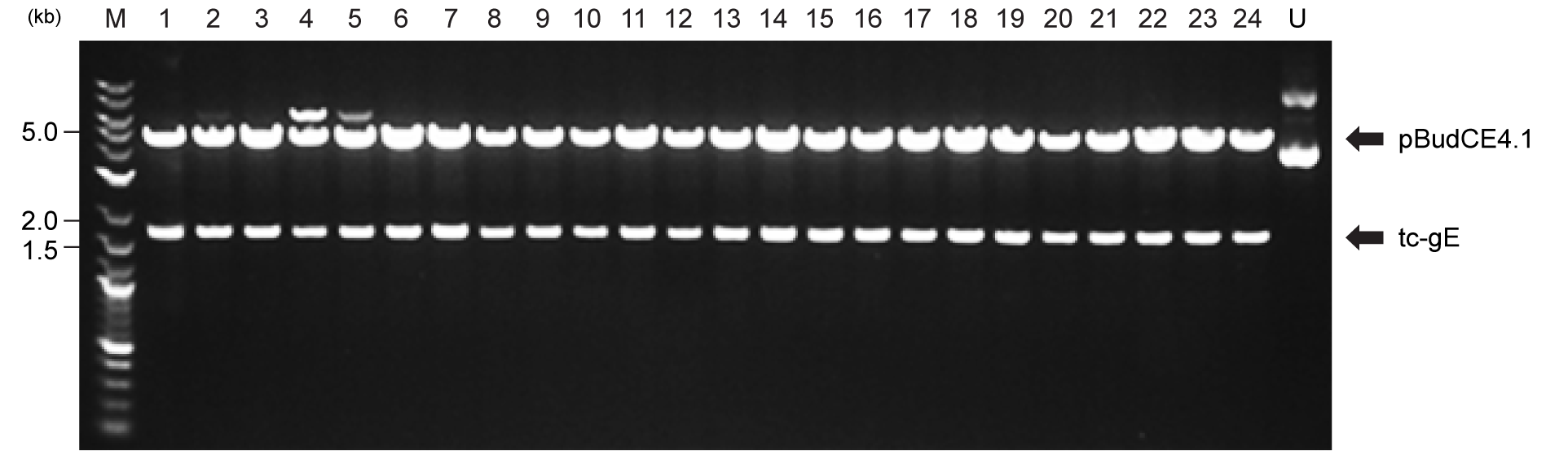

Supplement: Supplementary file 1 — Supporting information [file IID3-8-216-s004.tif]

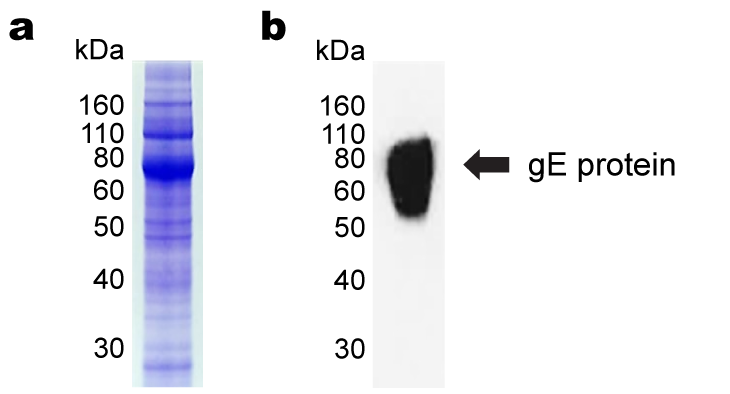

Supplement: Supplementary file 2 — Supporting information [file IID3-8-216-s003.tif]

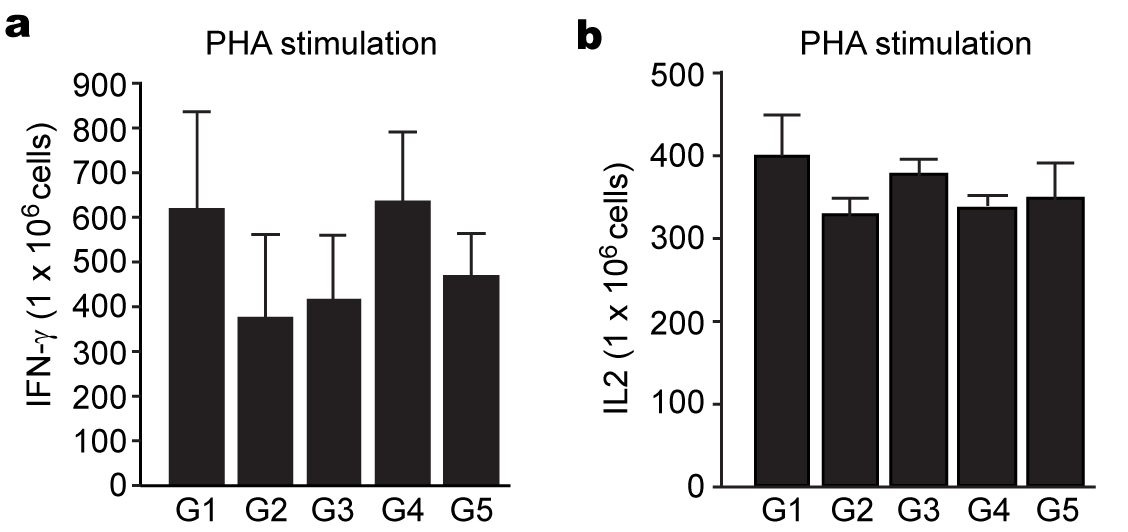

Supplement: Supplementary file 3 — Supporting information [file IID3-8-216-s005.tif]

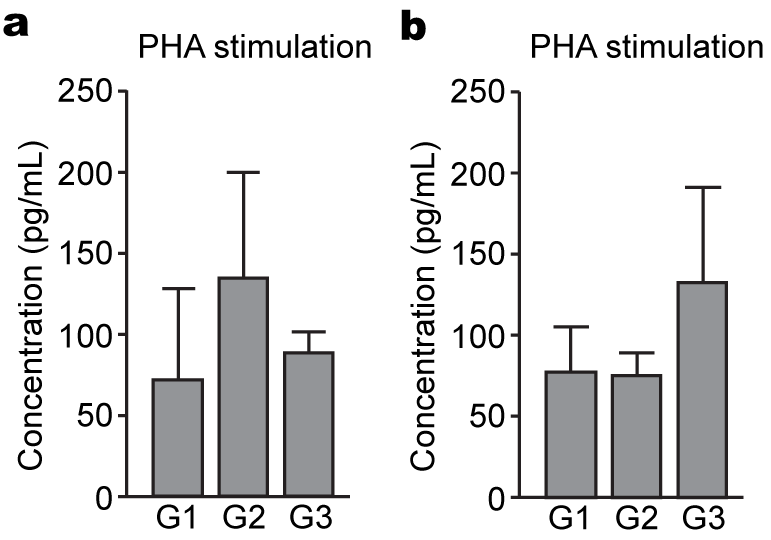

Supplement: Supplementary file 4 — Supporting information [file IID3-8-216-s001.tif]
