# Supplementary material for: Evaluation of glycoprotein E subunit and live attenuated varicella‐zoster virus vaccines formulated with a single‐strand RNA‐based adjuvant
Source: Immun Inflamm Dis. 2020 Mar 13;8(2):216–27. doi: 10.1002/iid3.297 (PMC7212201; doi:10.1002/iid3.297)
Supplement: Supplementary file 5 — Supporting information [file IID3-8-216-s002.docx]

***JEEN LEE*** et al***.***

Evaluation of glycoprotein E subunit and live attenuated varicella-zoster virus vaccines formulated with a single-strand RNA-based adjuvant

Su Jeen Lee[^a^](#MEP_L_aff1)^,^[^b^](#MEP_L_aff2)^,^^[[1]](#footnote-1)^, Hyo-Jung Park[^a^](#MEP_L_aff1)^†^, Hae Li Ko[^a^](#MEP_L_aff1)^†^, Jung Eun Lee[^b^](#MEP_L_aff2), Hyun Joo Lee[^b^](#MEP_L_aff2), Hun Kim[^b^](#MEP_L_aff2)*,* and Jae-Hwan Nam[^a^](#MEP_L_aff1)[^*^](#MEP_L_cor3)

^a^Department of Biotechnology, The Catholic University of Korea, Bucheon 14662, Republic of Korea

^b^Department of R&D, SK bioscience, Pangyoro 332, Bundang-gu 13494, Republic of Korea

**^*^Correspondence** Jae-Hwan Nam, Ph.D., Department of Biotechnology, The Catholic University of Korea, 43-1 Yeokgok-dong, Wonmi-gu, Bucheon 14662, Republic of Korea. E-mail: jhnam@catholic.ac.kr

**ABSTRACT**

Varicella-zoster virus (VZV), a human alphaherpesvirus 3, elicits both chickenpox and shingles and/or postherpetic neuralgia. A live attenuated vaccine (LAV) and glycoprotein E (gE) subunit vaccine were developed to prevent VZV-induced diseases. We recently reported that single-strand (ss) RNA based on the intergenic region of the internal ribosome entry site of cricket paralysis virus (CrPV) is an effective adjuvant for protein-based and virus-like particle-based vaccines. Here, we evaluated two new types of VZV vaccines: a gE subunit vaccine produced by a CHO expression system and an LAV from Oka/SK strains. We further appraised the adjuvant effect of the same CrPV ssRNA encoding the gE gene formulated in the two vaccines using VZV-primed C57BL/6 mice and guinea pigs. The gE subunit vaccine induced gE-specific antibodies and CD4+ T cell responses (indicated by IFN-γ and IL-2 secretion) in the ssRNA-based adjuvant containing the VZV gE gene. Therefore, an ssRNA adjuvant combined with gE antigen can trigger the innate immune response and induce an adaptive immune response to ultimately activate humoral and cell-mediated responses. VZV LAV could also induce VZV-specific antibodies and IFN-γ stimulated by LAV, whereas the effect of ssRNA as a vaccine adjuvant could not be confirmed. However, the ssRNA adjuvant increased VZV-specific neutralizing antibody response. Taken together, these results highlight that the gE subunit vaccine and LAV developed in this study can be functional VZV vaccines, and ssRNAs appear to function better as adjuvants in a subunit vaccine than in an LAV.

Graphical Abstract

We evaluated two new varicella-zoster virus (VSV) vaccines, a glycoprotein E (gE) subunit-based vaccine and live attenuated vaccine, that were formulating with ssRNA as a potential adjuvant. The gE subunit vaccine and LAV developed in this study can be functional VZV vaccines, and ssRNAs appear to function better as adjuvants in a subunit vaccine than in an LAV.

fx1

**Key words**

chickenpox; gE subunit vaccine; live attenuated vaccine; RNA adjuvant; shingles; varicella-zoster virus

**List of Abbreviations**

APCs antigen presenting cells

CHO Chinese hamster ovary

CrPV cricket paralysis virus

DPBS Dulbecco's phosphate-buffered saline

DCs dendritic cells

ELISA enzyme-linked immunosorbent assay

ELISPOT Enzyme-linked immune absorbent spot

FAMA Fluorescent antibody to membrane antigen

GAPDH glyceraldehyde-3-phosphate dehydrogenase

gE glycoprotein E

IGR intergenic region

IFN interferon

IL interleukin

IRES internal ribosome entry site

LAV live attenuated vaccine

PBS phosphate-buffered saline

PHA phytohemagglutinin

PFU plaque forming unit

RT room temperature

SD standard deviations

SDS-PAGE sodium dodecyl sulfate polyacrylamide gel electrophoresis

ssRNA single-strand RNA

TLR Toll-like receptor

TM transmembrane

TMB tetramethylbenzidine

UF/DF ultrafiltration/diafiltration

VZV Varicella-zoster virus

WVSS working virus seed stock

# **INTRODUCTION**

Varicella-zoster virus (VZV) induces chickenpox (varicella), shingles (herpes zoster), and/or postherpetic neuralgia. Varicella is the primary VZV infection and it occurs most frequently in children. Herpes zoster occurs mainly in adults or immunocompromised hosts as a consequence of latent VZV reactivation (1). VZV is a member of the human herpesvirus family encoding five major glycoproteins designated gpI–gpV (2). Glycoproteins are critical factors for VZV entry and replication. Thus, they are attractive targets for antiviral drug development (3). VZV gE among VZV glycoproteins is the most abundant and immunogenic. It participates in viral replication and cell-cell transmission. Moreover, it contains B cell and CD4+ T cell epitopes and elicits complement-dependent neutralizing antibodies and cell-mediated immunity (4). VZV-specific CD4+ T cells synthesize Th1-like cytokines such as interleukin-2 (IL-2) and interferon (IFN)-γ. They induce major histocompatibility complex class II-restricted cytotoxicity (5,6). Therefore, CD4+ T cells expressing IL-2 and IFN-γ were selected as immune markers to evaluate cell-mediated immune responses to VZV vaccines (4,7-9). VZV gE is an attractive candidate for the development of VZV subunit vaccines because the VZV gE antigen, also known as CD4+ T-cell antigen, is capable of inducing both humoral and cell-mediated immune responses (10-13).

Vaccines currently used to prevent VZV include live attenuated varicella vaccine (LAV) developed by Takahashi and colleagues in 1974 (14) and several other varicella vaccines licensed in several countries. The herpes zoster LAVs, ZOSTAVAX (Merck & Co., Inc., Darmstadt, Germany) and SKYZoster (SK bioscience Co. Ltd., Andong, Korea), have been licensed. LAV has preventive efficacy against varicella in the range of 70–96%. In contrast, its preventive efficacy against herpes zoster is only ~60% (15). LAV promotes relatively lower VZV-specific cellular immune responses against herpes zoster in older patients as immunosenescence accompanies the aging process. Immunosenescence is characterized by decreased T cell numbers and impaired T cell function (16). A subunit vaccine comprising VZV gE and the liposome-based adjuvant Shingrix (GlaxoSmithKline Biologicals, Rixensart, Belgium) was licensed in 2017. Its reported efficacy against herpes zoster was 97% (15). Therefore, the subunit vaccine may have greater efficacy against herpes zoster than LAV. A recombinant subunit vaccine is a potential alternative to live attenuated herpes zoster vaccine. However, the low immunogenicity of individual viral proteins may have to be enhanced with adjuvants (17).

Adjuvants are immunomodulating substances that may be combined in formulations to increase their immunostimulatory efficacy (18). Certain adjuvants have been approved for clinical use. All new adjuvants should be compared with the gold-standard aluminum-based adjuvants (alum). Alum has been used to increase vaccine formulation efficacy for▒>▒90▒yr (19). However, there are still limitations of Alum that require supplementation to boost vaccine efficacy. For example, Alum typically induces a Th2 response, which mediates the differentiation of B cells that secrete Th2-cell-associated antibody isotypes, as opposed to inducing a very low Th1 response that is required to activate innate immune response (20,21), as a prerequisite to guarantee a more effective and/or long-term immune response (22). The ideal adjuvant induces innate immune responses, thereby activating adaptive immune responses (23,24). Toll-like receptor (TLR) agonists and oil-in-water emulsions have been developed to complement alum adjuvants (25,26). Our new candidate adjuvant is a single-strand (ss) RNA derived from the cricket paralysis virus (CrPV) intergenic region (IGR) of the internal ribosome entry site (IRES). It induces balanced Th1/Th2 responses, enhances innate immune response, and increases vaccine efficacy (27).

Here, we modified this novel ssRNA adjuvant to encode the VZV gE gene. The latter was then tested in a gE subunit vaccine expressed in a Chinese hamster ovary (CHO) cell culture platform and LAV bearing the Oka/SK strain. In VZV-primed mice, we assessed whether this ssRNA adjuvant induces humoral- and cell-mediated immunity in the VZV gE subunit vaccine. Adjuvants are not usually included in LAV formulations. However, we used guinea pigs to evaluate the ability of the ssRNA adjuvant to enhance neutralizing antibody production and cell-mediated immune response in VZV LAV. Based on all results, we showed the potential of ssRNA adjuvants to compensate for the limitations of protein-based vaccines with respect to low T cell activity and short-term responses, as well as to increase the neutralizing antibody in LAV for preventing VZV induced disease.

# **MATERIALS AND METHODS**

## ***VZV gE antigen***

A truncated form of the VZV gE antigen with a deleted transmembrane (TM) domain was used in this study. Gene cloning for the gE antigen was performed with a primer and a vector (pBudCE4.1) ([🏳](#MEP_L_tbl1)Table [1](#MEP_L_tbl1)) after isolating the DNA from Oka/SK WVSS (working virus seed stock). The VZV gE antigen was expressed in a CHO-K1 (ATCC CCL-61) cell line via a CHO transfection kit including Lipofectamine LTX-Plus (No. 15338-100; Invitrogen, Carlsbad, CA, USA). Single-cell colonies were obtained according to gE expression in isolated single cells. An enzyme-linked immunosorbent assay (ELISA) identified four stable cell clone candidates with high antigen expression that were adapted in serum-free media. After 50 serial passages, only one clone (15) was selected. Cells with confirmed gE antigen expression were cultured to 8▒L. The antigen was purified via serial ultrafiltration/diafiltration (UF/DF) steps and anion exchange chromatography and concentrated to 30▒kDa for immunization. UF/DF was performed on a 10-kDa Pellicon membrane (No. P2B010A01; Pellicon 2 Mini ultrafiltration module Biomax; EMD Millipore, Billerica, MA, USA). Samples were loaded onto a TMAE(M) column (MiniChrom Column Fractogel^®^ TMAE (M); Merck, Darmstadt, Germany) and eluted with 250▒mM NaCl in 20▒mM piperazine buffer. The eluted samples were concentrated in a 30-kDa Centricon^®^ Plus-70 centrifugal filter (EMD Millipore, Billerica, MA, USA). The final mass of the total antigen after concentration and purification was 3▒mg.

## ***Preparation of the RNA adjuvant***

### *DNA template*

The RNA platform was designed with CrPV IGR IRES and SV40 late-polyadenylation signal sequences (28). The RNA platform consisted of four restriction enzyme sequences and a multicloning site between the untranslated regions to permit the insertion of the VZV ORF68 (gE) gene.

### *In vitro transcription and RNA purification*

The DNA platform was designed with CrPV IGR IRES and SV40 late-polyadenylation signal sequences (28). DNA templates were linearized with *Not*I. *In vitro* transcription was performed with an EZ^TM^ T7 high-yield *in vitro* transcription kit (Enzynomics, Seoul, Korea). Three micrograms of linearized DNA template were incubated with T7 transcription buffer, MgCl_2_, 10▒mM dithiothreitol, enhancer solution, 5▒mM rNTP, nuclease-free water, and 200 units T7 enzyme mix for 1▒h at 37▒°C. The transcripts were incubated with RNase-free DNase I (Promega, Madison, WI, USA) for 15▒min at 37▒°C. The reaction was terminated by incubation at 65▒°C for 10▒min. RNA was purified by the LiCl method. RNA purity and concentration were evaluated with a NanoDrop 2000 spectrophotometer (Thermo Fisher Scientific, Waltham, MA, USA). RNA integrity was evaluated by denaturing gel electrophoresis.

### *Immunoblot analysis*

A549 cells were cultured in a six-well plate for 12▒h at a density of 5▒×▒10^5^ well^-1^ in medium free of 10% (v/v) fetal bovine serum. The cells were then stimulated with 2▒μg ssRNA adjuvant for 24▒h, lysed by vortexing at 10-min intervals for 1▒h in radioimmunoprecipitation assay buffer containing halt protease and phosphatase inhibitor cocktail, and centrifuged at 15,000▒g for 30▒min. The resulting supernatant was used as whole-cell lysate. Fifty micrograms protein was loaded onto sodium dodecyl sulfate polyacrylamide gel electrophoresis (SDS-PAGE) gel and electrophoretically transferred to a polyvinylidene fluoride membrane. The membrane was incubated for 12▒h with the indicated VZV-antibody (CHA Biotech, Seoul, Korea) and then incubated for 2▒h with horseradish peroxidase-conjugated goat anti-mouse antibody. The protein band of interest was visualized with a ChemiDoc imaging system (Bio-Rad Laboratories, Hercules, CA, USA). Equal protein loading was verified by glyceraldehyde-3-phosphate dehydrogenase (GAPDH) immunoblotting.

## ***Immunization***

Six-week-old C57BL/6 mice were primed with VZV bulk (Oka/SK; SK bioscience Co. Ltd., Andong, Korea) at a dose of ~2,000 PFU mouse^-1^. Thirty-five days after priming, VZV gE protein (10▒μg VZV antigen mouse^-1^) formulated with 20▒μg ssRNA adjuvant was injected twice into the upper thigh muscles at 4-wk intervals between inoculations. The mice were immunized in the same way with AddaVax (Cat. No. vac-adx-10; 10▒μg; InvivoGen, San Diego, CA, USA) as a reference control. Five groups were designated as follows: negative control (G1); LAV priming (G2); gE antigen (G3); AddaVax (G4); and ssRNA adjuvant (G5).

Six-week-old Dunkin-Hartley guinea pigs were primed with VZV bulk (Oka/SK; SK bioscience Co. Ltd., Andong, Korea) at a dose of ~5,000 PFU guinea pig^-1^. Thirty-five days after priming, the guinea pigs were subcutaneously injected twice with a human dose (0.5▒mL) of live attenuated herpes zoster vaccine (SKYZoster) with or without ssRNA adjuvant (50▒μg) at 2-wk intervals between inoculations. Three groups were designated as follows: negative control (G1); LAV (G2); and ssRNA adjuvant (G3).

## ***Immunoglobulin (Ig) ELISA***

VZV-specific total IgG, IgG1, and IgG2a in mouse serum and total IgG, IgG1, and IgG2 in guinea pig serum were measured by enzyme-linked immunosorbent assay (ELISA). The 96-well plates (Nunc Maxisorp^TM^; Thermo Fisher Scientific, Waltham, MA, USA) were coated with 50▒ng well^-1^ VZV gE for mice and 1,000 PFU well^-1^ VZV for guinea pigs and incubated overnight at 4▒°C. The wells were then blocked with 200▒μL of 5% (v/v) skim milk for 1▒h at room temperature (RT). Diluted serum samples and VZV gE Ab (No. 127-10031; RayBiotech, Inc., Peachtree Corners, GA, USA) were added to the plates and incubated for 2▒h at RT. The wells were then washed thrice with 200▒μL phosphate-buffered saline (PBS) mixed with 0.05% (v/v) Tween 20 (PBST). The following antibodies were then added: anti-mouse IgG (ab97265; Abcam, Cambridge, UK), IgG1 (ab97240; Abcam, Cambridge, UK), and IgG2a Ab (ab97245; Abcam, Cambridge, UK) or anti-guinea pig IgG (ab9608; Abcam, Cambridge, UK), IgG1 (ABIN457757; Antibodies, Cambridge, UK), and IgG2 Ab (GAGp/IgG2/PO; Nordic MUbio, Susteren, The Netherlands). The mixtures were then incubated for 1▒h at 37▒°C. After washing, 3,3’,5’5’-tetramethylbenzidine (TMB) substrate was added to the wells and the mixtures were incubated for 15▒min. A stop solution was then added to halt the reaction. Optical densities were measured at 450▒nm in a microplate reader.

## ***Enzyme-linked immune absorbent spot (ELISPOT) assay***

The spleen from a mouse immunized with VZV gE antigen was washed with RPMI media and lysed with ACK lysing buffer. Complete media were then added to the sample, the suspension was centrifuged for 5▒min at 400▒g, and the pellet was washed with PBS and recentrifuged to obtain the splenocytes. One hundred microliters of the cells were loaded in each well (10^6^ cells per well). The stimulation factor phytohemagglutinin (PHA; L89025; 5▒μg▒mL^-1^; Sigma-Aldrich Corp., St. Louis, MO, USA) served as a positive control. Then gE protein (5▒μg▒mL^-1^; SK bioscience, Co. Ltd., Andong, Korea) and Pepmix VZV gE (1.25▒μg▒mL^-1^; PM-VZV-gE; JPT Peptide Technologies, Berlin, Germany) were added and the suspension was incubated for 20▒h at 37▒°C. The cells were washed with PBST and anti-murine IFN-γ and IL-2 biotin detection Ab (ImmunoSpot^®^ murine IFN-γ and IL-2 single-color enzymatic ELISPOT kit; Cellular Technology Ltd., Shaker Heights, OH, USA) were added to each well. The plate was incubated for 2▒h at RT and washed thrice with 200▒μL PBST. STREP-Ab solution from the aforementioned kit was diluted to 1:1,000. Then 80▒μL of this dilution was added to each well and the suspensions were incubated for 30▒min at RT. Eighty microliters of diluted substrate solution was then added to each well. The wells were rinsed with distilled water to stop the reaction, the plates were allowed to air-dry, and the spots were counted.

## ***Cytokine ELISA***

Cultured guinea pig splenocytes (10^6^) harvested at 18 d after the second immunization with live VZV bulk containing Oka/SK strains and ssRNA adjuvant were mixed with live virus (1,000 plaque forming unit, PFU) and PHA (5▒μg▒mL^-1^). The cells were incubated for 19▒h at 37▒°C and plated in a 96-well plate coated with antibodies against IFN-γ (Guinea pig interferon gamma ELISA kit; abx051108; Abbexa Ltd., Cambridge, UK) or IL-2 (Guinea pig interleukin 2 (IL-2) ELISA kit; abx150425; Abbexa Ltd., Cambridge, UK). The standards and samples were added to the wells and the suspensions were incubated and washed with wash buffer in kit. One hundred microliters of biotin-conjugated antibody against IFN-γ was used for detection. After washing, 90▒μL TMB substrate was added to the wells. The suspensions were incubated for 30▒min at 37▒°C to visualize horseradish peroxidase activity. To stop the reaction, 50▒μL stop solution was added to each well. Optical densities were measured in a microplate reader at 450▒nm to calculate the IFN-γ or IL-2 concentration.

## ***Fluorescent antibody to membrane antigen (FAMA) assay***

FAMA was used to measure the production of neutralizing antibodies to VZV. To determine the anti-VZV IgG level, 30▒μL Dulbecco's phosphate-buffered saline (DPBS) was added to U-bottom 96-well plates. Guinea pig serum was serially diluted from 1:2 to 1:1,024. Cell-associated virus (30▒μL) from infected cells was added to the wells and the suspensions were incubated for 30▒min at 37▒°C. After centrifugation at 2,000▒rpm for 5▒min, the supernatant was removed and the cells were washed with 1% (v/v) gelatin-DPBS (2:1) buffer. Then 30▒μL of a 1:200 dilution of anti-GP IgG-FITC conjugate was added to each well and the suspensions were incubated for 30▒min at 37▒°C. After washing with 1% (v/v) gelatin-DPBS buffer, 4▒μL glycerol-DPBS (2:1) was added to each well and the suspensions were visualized by fluorescence microscopy.

## ***Statistical analysis***

All histomorphometry data are expressed as means▒±▒standard deviations (SD). One-way ANOVA was run to compare group means. A Levene test was performed to evaluate variance homogeneity. If no significant deviations were found, the data were then analyzed by a least significant difference test. If the Levene test detected significant deviations from variance homogeneity, the data were then assessed by a Bonferroni test. Statistical analyses were conducted in SPSS v. 14.0 for Windows (release 14.0▒K; IBM Corp., Armonk, NY, USA). Student’s *t*-test was run to identify differences between groups with large errors. Differences were considered significant at *P*▒<▒0.05.

# **RESULTS**

## ***VZV gE antigen preparation***

To prepare the VZV gE antigen for mouse immunization, we isolated the ORF68 gene (gE▒+▒gpI) from Oka/SK WVSS (SK bioscience Co. Ltd., Andong, Korea). The full-length gE gene was 1,872▒bp and it encoded 623 amino acids. However, the truncated form used for gE antigen expression here was 1,632▒bp and it encoded 544 amino acids. In the latter case, the transmembrane and the cytoplasmic tail (CT) of the *C*-terminal were deleted ([🏳](#MEP_L_fig1)Figure [1](#MEP_L_fig1)(a)) (29). The cloned truncated gE genes (Figure [1](#MEP_L_fig1)(b) and Supplementary Figure 1) were expressed in CHO-K1 cells. A western blot of the purified VZV gE proteins confirmed expression of clones 4, 5, 11, and 15 in the first single cells ([🏳](#MEP_L_fig2)Figure [2](#MEP_L_fig2)(a) and Supplementary Figure 2) and in 14 clones derived from clone 5 (Figure [2](#MEP_L_fig2)(b)). ELISA revealed that clones 10, 12, 13, 14, and 15 had high antigen expression (Figure [2](#MEP_L_fig2)(c) and 2(d)). Thus, they were selected for suspension culture adaptation in serum-free medium.

## ***LAV preparation***

The live attenuated vaccine (LAV) used to immunize the guinea pigs was manufactured by SK bioscience Co. Ltd. (Andong, Korea). It was approved in 2017 under the name SKYZoster and prescribed as a prophylactic against shingles (zoster) for adults aged▒≥▒50▒yr. Its active ingredient is Oka/SK strain and the other constituents include stabilizers such as gelatin, sucrose, and urea. The master- and working virus seeds (Oka/SK) were established with the attenuated Oka vaccine strain. Vaccine safety and immunogenicity were validated in clinical phase II/III trials and compared against Zostavax (Merck & CO. INC., Whitehouse Station, NJ08889, USA) (30). One lot from the commercial products (▒≥▒27,400 PFU 0.5▒mL^-1^) manufactured by SK bioscience Co. Ltd. (Andong, Korea) was used for the immunization here.

## ***Humoral immune response against VZV gE antigen in mice***

The ssRNA adjuvant was prepared using the RNA platform derived from CrPV IGR IRES ([🏳](#MEP_L_fig3)Figure [3](#MEP_L_fig3)(a)) and the VZV ORF68 gene (gE). *PacI-SacI* was used in the gene of interest (Figure [3](#MEP_L_fig3)(b)). Western blot with gE antibody (CHA Biotech, Seoul, Korea) confirmed that the ssRNA adjuvant with VZV ORF68 gene expressed 68-kDa gE protein in transfected A549 cells (Figure [3](#MEP_L_fig3)(c)).

To confirm the immune responses in mice subjected to the ssRNA adjuvant with the VZV gE gene, mouse blood was sampled for IgG detection at 5 d before the first immunization and again at 42 d and 58 d after the first immunization ([🏳](#MEP_L_fig4)Figure [4](#MEP_L_fig4)(a)). Total IgG, IgG1, and IgG2a titers were all similar at Days 42 and 58 for nearly all mouse groups. The VZV gE total IgG antibody titers were higher in G5 (with ssRNA adjuvant) than G3 (without ssRNA adjuvant) (Figure [4](#MEP_L_fig4)(b)). The IgG1 titers indicating a Th2 response were similar for G5 and G3 (Figure [4](#MEP_L_fig4)(c)) whereas the IgG2a titer indicating a Th1 response for G5 was about 10-fold higher than that for G3 (Figure [4](#MEP_L_fig4)(d)). G4 (with AddaVax) presented with the highest IgG1 and IgG2a titers (Figures [4](#MEP_L_fig4)(c) and 4(d)).

***CD4+ T cell-mediated immune response against VZV gE antigen in mice***

VZV-specific cytokines (IFN-γ and IL-2; also known as CD4+ T cell cytokines) (5) were measured by ELISPOT assay to confirm enhancement of the VZV-specific cellular immune response (31,32) with splenocytes from immunized mice at day 58 after the first immunization. The numbers of IFN-γ- and IL-2-secreting cells in the cultured splenocytes had increased in G5 (with ssRNA adjuvant) compared to those in G3 (without ssRNA adjuvant) after stimulation with gE protein and gE-specific peptide mixture (Pepmix) ([🏳](#MEP_L_fig5)Figure [5](#MEP_L_fig5)(a–d)). Moreover, the numbers of cells secreting IFN-γ and IL-2 were similar in G5 (with ssRNA adjuvant) and G4 (with Addavax) used as the reference control (Figure [5](#MEP_L_fig5)(a–d)). The numbers of IFN-γ- and IL-2-secreting cells after PHA stimulation (as a positive control) were dramatically increased in all groups (Supplementary Figure 3(a) and (b)).

***Humoral and CD4+ T cell-mediated immune responses against LAV for VZV in guinea pig***

We tested the effects of ssRNA adjuvant in LAV SKYZoster (SK bioscience Co. Ltd., Andong, Korea) which was approved for administration as a shingles vaccine. Blood samples were obtained at 5 d before the first immunization and again at 14 d and 32 d after the first immunization ([🏳](#MEP_L_fig6)Figure [6](#MEP_L_fig6)(a)). Total IgG, IgG1, and IgG2 titers were not significantly increased in G3 (with ssRNA adjuvant) relative to those in G2 (without ssRNA adjuvant) (Figure [6](#MEP_L_fig6)(b–d)). Thus, the ssRNA adjuvant had no influence on the live vaccine. IgG1 (Th2 immune response) showed a weak titer at 14 d after the first immunization but was dramatically increased after the second immunization at Day 32 in all groups (Figure [6](#MEP_L_fig6)(c)).

CD4+ T cell-mediated immune response was assessed by measuring IFN-γ and IL-2 in cultured splenocyte supernatants after VZV stimulation. The VZV-induced cytokines IFN-γ and IL-2 were slightly increased in G2 (without ssRNA adjuvant) and G3 (with ssRNA adjuvant) compared to that in G1 (negative control). However, the cytokine levels were not significantly different between G2 and G3 (Figure [6](#MEP_L_fig6)(e) and 6(f)). As a positive control, the numbers of IFN-γ-secreting cells after PHA stimulation were increased in all groups (Supplementary Figure 4(a)). But the numbers of IL-2-secreting cells were not increased significantly after PHA stimulation (Supplementary Figure 4(b)). Therefore, the ssRNA adjuvant was ineffective in LAV.

## ***Neutralizing antibodies against VZV in guinea pigs***

VZV-specific neutralizing antibodies were measured by FAMA. The titers in G3 (with ssRNA adjuvant) were about 2-fold higher than those in G2 (without ssRNA adjuvant) ([🏳](#MEP_L_fig7)Figure [7](#MEP_L_fig7)(a) and 7(b)). Therefore, the ssRNA adjuvant induced neutralizing antibodies and, by extension, a humoral immune response in the LAV to the same level as the protein subunit vaccine even without corresponding increases in ELISA antibodies or cytokines.

# **DISCUSSION**

ssRNA is recognized by TLR7 and/or TLR8, which are highly expressed in antigen presenting cells (APCs) to stimulate an adaptive immune response for antigen-specific T cells, accompanied by high-affinity antibody production (25). Despite this potential adjuvant effect, to date, RNA has not been applied in conventional vaccines. Here, we produced a VZV gE subunit vaccine and a LAV containing the Oka/SK strain and integrated our recently developed CrPV IGR IRES-derived ssRNA adjuvant encoding the VZV gE gene into them.

Mice immunized with the subunit vaccine containing the ssRNA adjuvant responded with IgG1 (Th2 response) and IgG2a (Th1 response) titers that were higher than those for the groups without ssRNA adjuvant. The numbers of IFN-γ- and IL-2-secreting cells were 2–3-fold higher in the ssRNA adjuvant-immunized groups than in those immunized with VZV gE antigen alone. IFN-γ and IL-2 activate Th1 cells that enhance proinflammatory, cell-mediated immunity (33). Increases of the IL-2-secreting immune cells indicates elevated T cell activation, expansion, differentiation, and maintenance. It also indicates differentiation of CD8+ T cells into terminal effector- and memory cells (34,35). Induction of VZV-specific T cell activation with ssRNA adjuvant is essential for the development of VZV vaccines for administration to older patients because T cell activity declines with age (4). Thus, ssRNA adjuvant may play an important role in VZV-specific T cell immunity and enhance cell-mediated immune response to vaccination.

Our data demonstrated that ssRNA adjuvant boosts gE antigen immunogenicity and augments gE-specific antibody and CD4+ T cell responses. Therefore, it enhances cell-mediated- and humoral immune responses. These findings corroborate those of a previous study in which ssRNA adjuvant was formulated with Middle East respiratory syndrome coronavirus spike protein (27). Protein-based vaccines are relatively less immunogenic because the potency of T cell activation is low (36). Thus, the ssRNA adjuvants must compensate for these deficiencies. The gE protein produced from the CHO-K1 cell expression system induced VZV gE-specific antibody. Thus, it is a good candidate VZV protein vaccine.

The ssRNA adjuvant in LAV (SKYZoster) did not increase humoral- or cellular immune responses in guinea pigs. LAVs can generally initiate innate immunity in dendritic cells (DCs). DCs induce innate inflammatory responses to pathogens in a manner similar to responses to viral infections. These innate immunity responses drive subsequent adaptive responses such as memory T cell and B cell activation (37-39). Adjuvants may function as pathogen-associated molecular patterns that trigger innate immune responses via different mechanisms including APC activation and maturation and the initiation of downstream adaptive immune activity (40). In an earlier study, we showed that ssRNA adjuvant induced immune response-related genes similar to those induced by LAV (27). Therefore, elicitation of the innate and adaptive immune responses by ssRNA adjuvant may be offset by the effects of VZV LAV.

The neutralizing antibodies measured by FAMA were about 2-fold higher in the groups receiving the ssRNA adjuvant in VZV LAV than in those that did not receive it despite the lack of difference in ELISA antibody (IgG1 and IgG2a) titers or Th1-related cytokine induction. The ssRNA adjuvant must recruit comparatively more APCs (especially DCs) at the injection site (27) and the resultant increase in APCs may present specific epitopes to B cells. However, further research is needed to validate this hypothesis.

AddaVax was the reference control vaccine used in this study. It resembles MF59 and is a squalene-based oil-in-water nanoemulsion that enhances cellular- and humoral immune responses. In contrast, AlhydroGel (aluminum hydroxide gel) drives a Th2 response. AddaVax significantly increases antibody titers with more balanced Th1/Th2 responses than those obtained with alum (41). Here, the ssRNA adjuvant induced humoral and balanced Th1/Th2 immune responses even though its antibody and cytokine induction levels in the VZV gE protein subunit vaccine were lower than those for AddaVax. Nevertheless, the mode of action of AddaVax has not yet been elucidated (42,43). A previous study established that ssRNA adjuvants are relatively safe (44).

Taken together, these results verify that the gE protein produced in this study can be a new VZV protein vaccine candidate. Moreover, the ssRNA derived from CrPV IGR IRES encoding the gE gene, which could express gE in transfected cells, can effectively function as a vaccine adjuvant in a protein-based subunit vaccine by activating humoral and cell-mediated immune responses. These findings are in accordance with our previous report (27). In addition, we showed that the ssRNA adjuvant could boost the production of neutralizing antibodies even in an LAV. Thus, it is expected that this previously developed ssRNA adjuvant will be useful as an immune stimulator for various vaccine types, including protein-based and even live attenuated vaccines.

# Uncited references

[[1](#MEP_L_bib1)]; [[2](#MEP_L_bib2)]; [[3](#MEP_L_bib3)]; [[4](#MEP_L_bib4)]; [[5](#MEP_L_bib5)]; [[6](#MEP_L_bib6)]; [[7](#MEP_L_bib7)]; [[8](#MEP_L_bib8)]; [[9](#MEP_L_bib9)]; [[10](#MEP_L_bib10)]; [[11](#MEP_L_bib11)]; [[12](#MEP_L_bib12)]; [[13](#MEP_L_bib13)]; [[14](#MEP_L_bib14)]; [[15](#MEP_L_bib15)]; [[16](#MEP_L_bib16)]; [[17](#MEP_L_bib17)]; [[18](#MEP_L_bib18)]; [[19](#MEP_L_bib19)]; [[20](#MEP_L_bib20)]; [[21](#MEP_L_bib21)]; [[22](#MEP_L_bib22)]; [[23](#MEP_L_bib23)]; [[24](#MEP_L_bib24)]; [[25](#MEP_L_bib25)]; [[26](#MEP_L_bib26)]; [[27](#MEP_L_bib27)]; [[28](#MEP_L_bib28)]; [[29](#MEP_L_bib29)]; [[30](#MEP_L_bib30)]; [[31](#MEP_L_bib31)]; [[32](#MEP_L_bib32)]; [[33](#MEP_L_bib33)]; [[34](#MEP_L_bib34)]; [[35](#MEP_L_bib35)]; [[36](#MEP_L_bib36)]; [[37](#MEP_L_bib37)]; [[38](#MEP_L_bib38)]; [[39](#MEP_L_bib39)]; [[40](#MEP_L_bib40)]; [[41](#MEP_L_bib41)]; [[42](#MEP_L_bib42)]; [[43](#MEP_L_bib43)]; [[44](#MEP_L_bib44)]

ACKNOWLEDGMENTS

The authors thank the personnel of the Laboratory of Viral Immunology for their excellent technical assistance with the experimental animals.

**DATA AVAILABILITY STATEMENT**

All data are shown within the manuscript and figures. The raw data and the analysis details that support the findings of this study are available from the corresponding author upon reasonable request.

**ETHICS STATEMENT**

All immunization-related animal experiments in this study were conducted in accordance with the guidelines of the Institutional Animal Care and Use Committee of the QuBEST BIO (Approval No. QBSIACUC-A18090 for mouse / QBIACUC-A18024 for guinea pig).

**DISCLOSURE OF POTENTIAL CONFLICTS OF INTEREST**

The authors declare no competing interests.

**FUNDING**

This work was supported by the Korean Health Technology R&D Project through the Korea Health Industry Development Institute (KHIDI). It was funded by the Ministry of Health & Welfare, Republic of Korea (No. HI15C2955) and the Basic Science Research Program through the National Research Foundation (NRF) funded by the Ministry of Science, ICT & Future Planning (No. NRF-2015M3A9B5030157).

REFERENCES

☑1. Haumont M., Jacquet A., Massaer M., Deleersnyder V., Mazzu P., Bollen A., Jacobs P. (1996) Purification, characterization and immunogenicity of recombinant varicella-zoster virus glycoprotein gE secreted by Chinese hamster ovary cells. *Virus Res* **40**: 199-204. doi: 10.1016/0168-1702(95)01270-2.

🛇2. Vafai A. (1994) Antibody-binding sites on truncated forms of varicella-zoster virus gpI(gE) glycoprotein. *Vaccine* **12**:1265-9. doi: 10.1016/s0264-410x(94)80030-4.

🛇3. Oliver S.L., Yang E., Arvin A.M. (2016) Varicella-zoster virus glycoproteins: entry, replication, and pathogenesis. *Curr Clin Microbiol Rep* **3**: 204-15. doi: 10.1007/s40588-016-0044-4.

🛇4. Dendouga N., Fochesato M., Lockman L., Mossman S., Giannini S.L. (2012) Cell-mediated immune responses to a varicella-zoster virus glycoprotein E vaccine using both a TLR agonist and QS21 in mice. *Vaccine* **30**: 3126-35. doi: 10.1016/j.vaccine.2012.01.088.

🛇5. Frey C.R., Sharp M.A., Min A.S., Schmid D.S., Loparev V., Arvin A.M. (2003) Identification of CD8+ T cell epitopes in the immediate early 62 protein (IE62) of varicella-zoster virus, and evaluation of frequency of CD8+ T cell response to IE62, by use of IE62 peptides after varicella vaccination. *J Infect Dis* **188**: 40-52. doi: 10.1086/375828.

🛇6. Jones L., Black A.P., Malavige G.N., Ogg G.S. (2006) Persistent high frequencies of varicella-zoster virus ORF4 protein-specific CD4+ T cells after primary infection. *J Virol* **80**: 9772-8. doi:10.1128/JVI.00564-06.

🛇7. Sadaoka K., Okamoto S., Gomi Y., Tanimoto T., Ishikawa T., Yoshikawa T., Asano Y., Yamanishi K., Mori Y. (2008) Measurement of varicella-zoster virus (VZV)-specific cell-mediated immunity: comparison between VZV skin test and interferon-γ enzyme-linked immunospot assay. *J Infect Dis* **198**: 1327-33. doi: 10.1086/592219.

🛇8. Sei J.J., Cox K.S., Dubey S.A., Antonello J.M., Krah D.L., Casimiro D.R., Vora K.A. (2015) Effector and central memory poly-functional CD4+ and CD8+ T cells are boosted upon ZOSTAVAX vaccination. *Front Immunol* **6**: 1-15. doi: 10.3389/fimmu.2015.00553.

🛇9. Fochesato M., Dendouga N., Boxus M. (2016) Comparative preclinical evaluation of AS01 versus other adjuvant systems in a candidate herpes zoster glycoprotein E subunit vaccine. *Hum Vaccin Immunother* **12**: 2092-5. http://dx.doi.org/10.1080/21645515.2016. 1154247.

🛇10. Leroux-Roels I., Leroux-Roels G., Clement F., Vandepapelière P., Vassilev V., Ledent E., Heineman T.C. (2012) A phase 1/2 clinical trial evaluating safety and immunogenicity of a varicella zoster glycoprotein E subunit vaccine candidate in young and older adults. *J Infect Dis* **206**: 1280-90. doi: 10.1093/infdis/jis497.

🛇11. Olson J.K., Santos R.A., Grose C. (1998) Varicella-zoster virus glycoprotein gE: endocytosis and trafficking of the Fc receptor. *J Infect Dis* **178**: S2-6. doi: 10.1086/514255.

☑12. Kimura H., Wang Y., Pesnicak L., Cohen J.I., Hooks J.J., Straus S.E., Williams R.K. (1998) Recombinant varicella-zoster virus glycoprotein E and I: Immunologic responses and clearance of virus in a guinea pig model of chronic uveitis. *J Infect Dis* **178**: 310-317. doi: 10.1086/515638.

🛇13. Laing K.J., Russell R.M., Dong L., Schmid D.S., Stern M., Magaret A., Haas J.G., Johnston C., Wald A., Koelle D.M. (2015) Zoster vaccination increases the breadth of CD4+ T cells responsive to varicella zoster virus. *J Infect Dis* **212**: 1022-31. doi:10.1093/infdis/jiv164.

🛇14. Gershon A.A., Breuer J., Cohen J.I., Cohrs R.J., Gershon M.D., Gilden D., Grose C., Hambleton S., Kennedy P.G.E., Oxman M.N., Seward J.F., Yamanishi K. (2017) Varicella zoster virus infection. *Nat Rev Dis Primers* **1**: 1-41. doi: 10.1038/nrdp.2015.16.

🛇15. Nelson C.S., Herold B.C., Permar S.R. (2018) A new era in cytomegalovirus vaccinology: considerations for rational design of next-generation vaccines to prevent congenital cytomegalovirus infection. *NPJ Vaccines* **38**: 1-9. doi: 10.1038/s41541-018-0074-4.

☑16. Chlibek R., Smetana J., Pauksens K., Rombo L., Van den Hoek J.A.R., Richardus J.H., Plassmann G., Schwarz T.F., Ledent E., Heineman T.C. (2014) Safety and immunogenicity of three different formulations of an adjuvanted varicella-zoster virus subunit candidate vaccine in older adults: A phase II, randomized, controlled study. *Vaccine* **32**: 1745-53. http://dx.doi.org/10.1016/j.vaccine.2014.01.019.

🛇17. Chlibek R.J., Bayas M., Collins H.M., Rodriguez de la Pinta L., Ledent E., Mols J.F., Heineman T.C. (2013) Safety and immunogenicity of an AS01-adjuvanted varicella-zoster virus subunit candidate vaccine against herpes zoster in adults▒≥▒50 years of age. *J Infect Dis* **208**: 1953-61. doi: 10.1093/infdis/jit365.

🛇18. Vandepapelière P., Horsmans Y., Moris P., Mechelen M.V., Janssens M., Koutsoukos M., Belle P.V., Clement F., Hanon E., Wettendorff M., Garçon N., Leroux-Roels G. (2008) Vaccine adjuvant systems containing monophosphoryl lipid A and QS21 induce strong and persistent humoral and T cell responses against hepatitis B surface antigen in healthy adult volunteers. *Vaccine* **26**: 1375-86. doi: 10.1016/j.vaccine.2007.12.038.

🛇19. HogenEsch H., O’Hagan D.T., Fox C.B. (2018) Optimizing the utilization of aluminum adjuvants in vaccines: you might just get what you want. *NPJ Vaccines* **51**: 1-11.

🛇20. Marrack P., McKee A.S., Munks M.W. (2009) Towards an understanding of the adjuvant action of aluminium. *Nat Rev Immunol* **9**: 287-93. doi:10.1038/nri2510.

☑21. He P., Zou Y., Hu Z. (2015) Advances in aluminum hydroxide-based adjuvant research and its mechanism. *Hum Vaccin Immunother* **11**: 477-88. http://dx.doi.org/10.1080/ 21645515.2014.1004026.

🛇22. Mian M.F., Ahmed A.N., Rad M., Babaian A., Bowdish D., Ashkar A.A. (2013) Length of dsRNA (poly I:C) drives distinct innate immune responses, depending on the cell type. *J Leukoc Biol* **94**: 1025-36. doi:10.1189/jlb.0312125.

🛇23. Leroux-Roels, G. (2010) Unmet needs in modern vaccinology: adjuvants to improve the immune response. *Vaccine* **28**: C25-36. https://doi.org/10.1016/j.vaccine.2010.07.021.

🛇24. Awate S., Babiuk L.A., Mutwiri G. (2013) Mechanisms of action of adjuvants. *Front Immunol* **4**: 1-10. doi: 10.3389/fimmu.2013.00114.

🛇25. Steinhagen F., Kinjo T., Bode C., Klinman D.M. (2011) TLR-based immune adjuvants. *Vaccine* **29**: 3341-55. https://doi.org/10.1016/j.vaccine.2010.08.002.

☑26. Coffman R.L., Sher A., Seder R.A. (2010) Vaccine adjuvants: putting innate immunity to work. *Immunity* **33**: 492-503. doi:10.1016/j.immuni.2010.10.002.

🛇27. Kwak H.W., Park H.J., Ko H.L., Park H., Cha M.H., Lee S.M., Kang K.W., Kim R.H., Ryu S.R., Kim H.J., Kim J.O., Song M., Kim H., Jeong D.G., Shin E.C., Nam J.H. (2019) Cricket paralysis virus internal ribosome entry site-derived RNA promotes conventional vaccine efficacy by enhancing a balanced Th1/Th2 response. *Vaccine* **37**: 5191-202. doi: 10.1016/j.vaccine.2019.07.070.

🛇28. Ko H.L., Park H.J., Kim J., Kim H., Youn H., Nam J.H. (2019) Development of an RNA expression platform controlled by viral internal ribosome entry sites. *J* *Microbiol Biotechnol* **29**: 127-40. doi: 10.4014/jmb.1811.11019.

🛇29. Thomsson E., Persson L., Grahn A., Snäll J., Ekblad M., Brunhage E., Svensson F., Jern C., Hansson G.C., Bäckström M., Bergström T. (2011) Recombinant glycoprotein E produced in mammalian cells in large-scale as an antigen for varicella-zoster-virus serology. *J Virol Method* **175**: 53-9. doi: 10.1016/j.jviromet.2011.04.014.

🛇30. Choi W.S., Choi J.H., Jung D.S., Choi H.J., Kim Y.S., Lee J., Jang H.C., Shin E.C., Park J.S., Kim H., Cheong H.J. (2019) Immunogenicity and safety of a new live attenuated herpes zoster vaccine (NBP608) compared to Zostavax^®^ in healthy adults aged 50 years and older. *Vaccine* **37**: 3605-10. doi: 10.1016/j.vaccine.2019.04.046.

🛇31. Weinberg A., Zhang J.H., Oxman M.N., Johnson G.R., Hayward A.R., Caulfield M.J., Irwin M.R., Clair J., Smith J.G., Stanley H., Marchese R.D., Harbeche R., Williams H.M., Chan I.S.F., Arbeit R.D., Gershon A.A., Schödel F., Morrison V.A., Kauffman C.A., Straus S.E., Schmader K.E., Davis L.E., Levin M.J. (2009) Varicella-zoster virus-specific immune responses to herpes zoster in elderly participants in a trial of a clinically effective zoster vaccine. *J Infect Dis* **200**: 1068-77. doi: 10.1086/605611.

🛇32. Smith J.G., Levin M., Vessey R., Chan I.S.F., Hayward A.R., Liu X., Kaufhold R.M., Clair J., Chalikonda I., Chan C., Bernard M., Wang W.W., Keller P., Caulfield M.J. (2003) Measurement of cell-mediated immunity with a varicella-zoster virus-specific interferon-γ ELISPOT assay: Responses in an elderly population receiving a booster immunization. *J Med Virol* **70**: S38-S41. doi: 10.1002/jmv.10318.

33. Broere F., Apasov S.G., Sitkovsky M.V., van Eden W. (2011) T cell subsets and T cell-mediated immunity. In: Nijkamp F., Parnham M., eds. Principles of Immunopharmacology, 3^rd^ edn. Basel, Switzerland: Birkhäuser Basel, pp. 15-27. doi: 10.1007/978-3-0346-0136-8_2.

🛇34. Dai Z., Konieczny B.T., Lakkis F.G. (2000) The dual role of IL-2 in the generation and maintenance of CD8+ memory T cells. *J Immunol* **165**: 3031-6. https://doi.org/10.4049/jimmunol.165.6.3031.

🛇35. Boyman O., Cho J.H., Sprent J. (2010) The role of interleukin-2 in memory CD8 cell differentiation. *Adv Exp Med Biol* **684**: 28-41. https://doi.org/10.5167/uzh-41847.

☑36. Martins K.A.O., Cooper C.L., Stronsky S.M., Norris S.L.W., Kwilas S.A., Steffens J.T., Benko J.W., van Tongeren S.A., Bavari S. (2016) Adjuvant-enhanced CD4 T cell responses are critical to durable vaccine immunity. *EbioMedicine* **3**: 67-78. http://dx.doi.org/10.1016/j.ebiom.2015.11.041.

🛇37. Hespel C., Moser M. (2012) Role of inflammatory dendritic cells in innate and adaptive immunity. *Eur J Immunol* **42**: 2535-43. doi: 10.1002/eji.201242480.

🛇38. Price J.D., Tarbell K.V. (2015) The role of dendritic cell subsets and innate immunity in the pathogenesis of type a diabetes and other autoimmune diseases. *Front Immunol* **6**: 1-12. doi: 10.3389/fimmu.2015.00288.

🛇39. Abendroth A., Morrow G., Cunningham A.L., Slobedman B. (2001) Varicella-zoster virus infection of human dendritic cells and transmission to T cells: Implications for virus dissemination in the host. *J Virol* **75**: 6183-92. doi: 10.1128/JVI.75.13.6183-6192.2001.

🛇40. Pasquale A.D., Preiss S., Da Silva F.T., Garçon N. (2015) Vaccine adjuvants: from 1920 to 2015 and beyond. *Vaccines* **3**: 320-43. doi: 10.3390/vaccines.3020320.

🛇41. Van Diepen M.T., Chapman R., Moore P.L., Margolin E., Hermanus T., Morris L., Ximba P., Rybicki E.P., Williamson A.L. (2018) The adjuvant AlhydroGel elicits higher antibody titres than AddaVax when combined with HIV-1 subtype C gp140 from CAP256. *PLoS One* **13**: 1-15. https://doi.org/10.1371/journal.pone.0208310.

🛇42. Choubini E., Habibi M., Khorshidi A., Ghasemi A., Karam M.R.A., Bouzari S. (2018) A novel multi-peptide subunit vaccine admixed with AddaVax adjuvant produces significant immunogenicity and protection against Proteus mirabilis urinary tract infection in mice model. *Mol Immunol* **96**: 88-97. https://doi.org/10.1016/j.molimm.2018.03.001.

🛇43. Sastry M., Zhang B., Chen M., Joyce M.G., Kong W.P., Chuang G.Y., Ko K., Kumar A., Silacci C., Thom M., Salazar A.M., Corti D., Lanzavecchia A., Taylor G., Mascola J.R., Graham B.S., Kwong P.D. (2017) Adjuvants and the vaccine response to the DS-Cav1-stabilized fusion glycoprotein of respiratory syncytial virus. *PLoS One* **12**: 1-21. https://doi.org/10.1371/journal.pone.0186854.

🛇44. Park H.J., Ko H.L., Won D.H., Hwang D.B., Shin Y.S., Kwak H.W., Kim H.J., Yun J.W., Nam J.H. (2019) Comprehensive analysis of the safety profile of a single-stranded RNA nano-structure adjuvant. *Pharmaceutics* **11**: 1-18. www.mdpi.com/journal/pharmaceutics.

TABLE 1. Primer sequences for expression vector cloning

| Vector | Primers | Sequence (5▒→▒3) | Enzyme |
| --- | --- | --- | --- |
| pBudCE4.1 | PB-gE_F | TCAGGTACCCGGACCATGGGGACAGTTAAT | *Kpn*I |
|  | PC-gE_R | ACCGGAGGGCTTGCATGAAATAAACTCGAG | *Xho*I |
|  | | | |

**Figure 1. Cloning of truncated VZV gE**. (a) Truncated VZV gE contained 544 of the 623 amino acids present in the full-length protein. In the former, the transmembrane (TM) and cytoplasmic tail (CT) regions were deleted. (b) Cloning expression vector (pBudCE4.1-tc-gE). Truncated VZV gE (1,632▒bp) was inserted into the pBudCE4.1 vector with *Kpn*I*-Xho*I. TM, Transmembrane domain.

**Figure 2. Confirmed VZV gE antigen expression (~70▒kDa) by clones in CHO-K1 cells**. (a) Clones 4, 5, 11, and 15) expressed gE antigen in first single-cell cloning. (b) Clones derived from clone 5 in (a) expressing gE antigen in second single-cell cloning. (c) ELISA results of 14 clones in (b). (d) 5 clones shown highest ELISA detection from (c). Lane numbers indicate each separately cultured clone.

**Figure 3. Structure of ssRNA adjuvant encoding VZV gE gene.** (a) Schematic of ssRNA adjuvant encoding VZV gE gene. (b) Denaturing gel electrophoresis of ssRNA adjuvant. (c) Immunoblot of VZV gE gene in A549 cells transfected with ssRNA adjuvant (2▒μg) for 24▒h.

**Figure 4. Humoral immune response of VZV-gE induced in C57BL/6 mice**. (a) Study design for gE protein-based vaccine immunization and schedules. All groups were inoculated twice at 4-wk interval. Sera at pre-injection (Day -5), 14 d (Day 42), and 30 d (Day 58) after second immunization were analyzed. Total IgG (b), IgG1 (c), and IgG2a (d) titers at –5 d, 42 d, and 58 d after first immunization. Data are means▒±▒SD (***P*▒<▒0.01, **P*▒<▒0.05 compared with LAV priming group G2). I.M., intramuscular

**Figure 5. Cytokine-secreting cell frequencies in immunized mouse splenocytes**. IFN-γ-secreting cell frequency in immunized mouse splenocytes after stimulation with gE protein (a) and Pepmix (b). IL-2-secreting cell frequency in immunized mouse splenocytes after stimulation with gE protein (c) and Pepmix (d). Data are means▒±▒SD (**P*▒<▒0.05). N.S., No Significance

**Figure 6. Humoral immune responses and IFN-γ and IL-2 levels in guinea pigs immunized with LAV**. (a) Study design for VZV LAV immunization and schedules. All groups were inoculated twice at 2-wk intervals. Sera at pre-injection (Day -5), 14 d, and 32 d after the first immunization were analyzed for total IgG (b), IgG1 (c), and IgG2 (d). IFN-γ (e) and IL-2 (f) levels in supernatants of cultured splenocytes after stimulation with LAV were measured by cytokine ELISA. Data are means▒±▒SD (***P*▒<▒0.01, **P*▒<▒0.05 compared with PBS immunization group G1).

**Figure 7. Neutralizing antibodies analyzed by FAMA to determine boosting effect of ssRNA adjuvants on VZV LAV**. Study design for VZV LAV immunization is shown in Fig. [6](#MEP_L_fig6)(a). Guinea pig serum was serially diluted from 1:2 to 1:1,024. Anti-GP IgG-FITC conjugate was used with 1:200. (a) Average FAMA titer was 186 for G2 (LAV alone) and 410 for G3 (LAV▒+▒ssRNA). (b) Cells were observed under fluorescence microscope.

**SUPPLEMENTARY FIGURE LEGENDS**

**Supplementary Figure 1. Gel running picture for truncated gE inserted in pBudCE4.1-tc-gE vector**. Vector (pBudCE4.1) was 4,595▒bp and truncated gE (tc-gE) was about 1.6▒kb.

**Supplementary Figure 2. Expression of VZV gE protein determined with (a) SDS-PAGE and (b) western blot using VZV gE-specific polyclonal antibody**.

**Supplementary Figure 3. The numbers of IFN-γ- and IL-2-secreting cells after PHA stimulation as a positive control. (a) IFN-γ and (b) IL-2 levels in mice**. Negative control (G1); LAV priming (G2); gE antigen (G3); AddaVax (G4); and ssRNA adjuvant (G5).

**Supplementary Figure 4. The numbers of IFN-γ- and IL-2-secreting cells after PHA stimulation as a positive control. (a) IFN-γ and (b) IL-2 levels in guinea pigs**. Negative control (G1); LAV (G2); and ssRNA adjuvant (G3).

1. These authors contributed equally. [↑](#footnote-ref-1)
